# Considerations on the taxonomy and morphology of *Microcotyle* spp.: redescription of *M. erythrini* van Beneden & Hesse, 1863 (*sensu stricto*) (Monogenea: Microcotylidae) and the description of a new species from *Dentex dentex* (L.) (Teleostei: Sparidae)

**DOI:** 10.1186/s13071-020-3878-9

**Published:** 2020-01-31

**Authors:** María Víllora-Montero, Ana Pérez-del-Olmo, Simona Georgieva, Juan Antonio Raga, Francisco Esteban Montero

**Affiliations:** Marine Zoology Unit, Cavanilles Institute of Biodiversity and Evolutionary Biology, Science Park, University of Valencia, C/Catedrático José Beltrán 2, 46980 Paterna, Spain

**Keywords:** *Microcotyle erythrini* (*sensu stricto*), *M. isyebi*, *M. whittingtoni* n. sp., Haptor morphology, Clamp morphology, Pseudocrypsis

## Abstract

**Background:**

*Microcotyle erythrini* van Beneden & Hesse, 1863 (Platyhelminthes: Monogenea) and other closely related species of the genus are often considered as cryptic. Records in hosts other than the type-host with no species confirmation by molecular analyses have contributed to this situation.

**Methods:**

Gill parasites of five sparid fishes, *Boops boops* (L.), *Pagellus erythrinus* (L.), *P. acarne* (Risso), *Dentex dentex* (L.) and *Pagrus pagrus* (L.), from the Western Mediterranean off Spain were collected. Specimens of *Microcotyle* spp. were characterised both molecularly and morphologically. Partial fragments (domains D1-D3) of the *28S* rRNA gene and the cytochrome *c* oxidase subunit 1 (*cox*1) gene were amplified and used for molecular identification and phylogenetic reconstruction. Principal components analysis was used to look for patterns of morphological separation.

**Results:**

Molecular analyses confirmed the identity of three species: *M. erythrini* ex *P. erythrinus* and *Pa. pagrus*; *M. isyebi* Bouguerche, Gey, Justine & Tazerouti, 2019 ex *B. boops*; and a species new to science described herein, *M. whittingtoni* n. sp. ex *D. dentex.* The specific morphological traits and confirmed hosts (*P. erythrinus* and *Pa. pagrus*) are delimited here in order to avoid misidentifications of *M. erythrini* (*sensu stricto*). *Microcotyle erythrini* (*s*.*s*.) is mostly differentiated by the shape of its haptor, which is also longer than in the other congeners. New morphological and molecular data are provided for *M. isyebi* from the Spanish Mediterranean enlarging the data on its geographical range. *Microcotyle whittingtoni* n. sp. is described from *D. dentex* and distinguished from the remaining currently recognised species of the genus by the number and robustness of the clamps.

**Conclusions:**

New diagnostic morphological traits useful to differentiate *Microcotyle* spp. are suggested: (i) haptor dimensions including lobes; (ii) the thickness of the clamps; (iii) the size and shape of spines of the genital atrium; (iv) the extension of the posterior extremities of vitelline fields; and (v) the shape of egg filaments. The use of new morphological approaches may allow considering these species of *Microcotyle* as being pseudocryptic. The use of representative undamaged specimens that have been genetically confirmed as conspecific is considered crucial to avoid abnormally wide morphological ranges that prevent species differentiation.
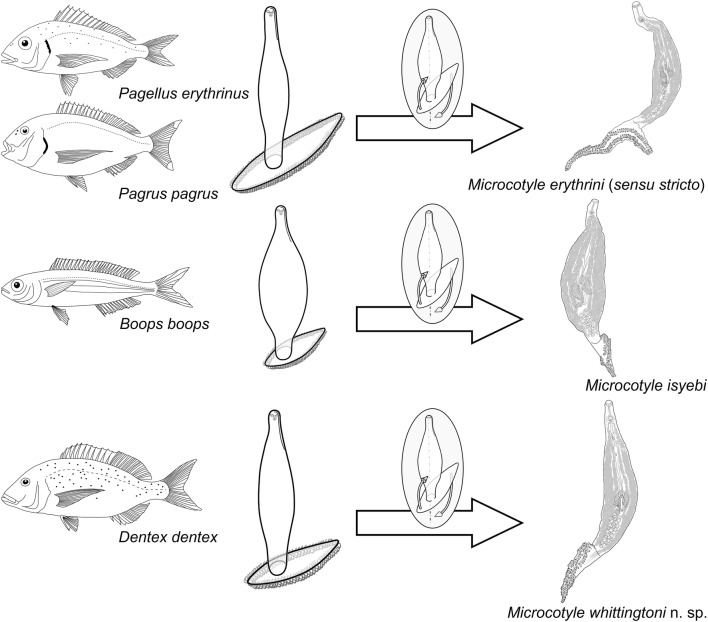

## Background

*Microcotyle erythrini* van Beneden & Hesse, 1863 (Monogenea: Microcotylidae) was originally described from *Pagellus erythrinus* (L.) (Teleostei: Sparidae) off the coast of Brest (France, North-East Atlantic) and to date it has been listed and considered a valid species [1–3]. Like many of the earliest descriptions of species of *Microcotyle*, *M. erythrini* was described briefly, and only differentiated by the number of clamps and testes, and the traits of the genital atrium [4]. Since the original description, many authors have recorded and described new specimens identified as *M. erythrini* in different sparid species, mostly in the Mediterranean Sea (see Table 1 in Bouguerche et al. [3], for details on the records of *M*. *erythrini*). These publications sometimes offered morphological ranges based on a combination of measurements of specimens from different host species (e.g. [5, 6]). Along this process, the morphological ranges of *M. erythrini* have been enlarged abnormally, which has made it difficult to define a clear and distinguishing morphology. Recently, with the help of molecular tools (*cox*1 partial fragment), *M*. *erythrini* has been split into two species, each in a different sparid host off the Algerian coast: *M. erythrini* ex *P. erythrinus* and *M. isyebi* Bouguerche, Gey, Justine & Tazerouti, 2019 ex *Boops boops* (L.) [3]. These authors also included the most recent morphometric information on *M. erythrini* from the type-host *P. erythrinus*. Bouguerche et al. [2, 3] suggested that morphological and molecular characterization of *M. erythrini*-like specimens infecting different sparid hosts would reveal higher parasite diversity.

The aim of the present study is a revision of the taxonomy of *Microcotyle* spp. in sparids from the Western Mediterranean off Spain. The specific objectives of the study are: (i) to describe a new species of *Microcotyle* parasitic in *Dentex dentex* (L.); (ii) to redescribe *M. erythrini* with the support of molecular evidence, define the actual morphological boundaries of the species and indicate the valid historical records; and (iii) to provide new morphological and molecular data useful for the taxonomy of *Microcotyle* spp. New morphological approaches and classification tools for species discrimination are proposed for these monogeneans which are notoriously difficult to differentiate.

## Methods

### Sample collection

A total of 150 fishes of four sparid species were examined for microcotylid infections: 40 bogues (*Boops boops*), 40 common pandoras (*Pagellus erythrinus*), 40 common dentexes (*Dentex dentex*) and 30 red porgies (*Pagrus pagrus*). Additionally, 40 axillary seabreams (*P. acarne* (Risso)) were also examined. Fishes were caught by commercial bottom trawling vessels during July of 2012 and 2013, off Guardamar del Segura, Alicante, Spain (38°05′N, 0°39′W; Western Mediterranean Sea, FAO fishing subarea 37.1). Fishes were transported on ice to the laboratory, where they were weighed, measured (weight provided in g and standard length in cm, expressed as the range with the mean and standard deviation (SD) in parentheses; only provided for infected hosts in the taxonomic summary) and then dissected for gill examination. Each pair of gills was dissected and inspected for parasites under a stereomicroscope. All parasites were collected and washed in 0.9% saline solution. For *Microcotyle* spp. specimens, two different protocols were used. Adult and completely mature specimens in optimal conditions (not broken, contracted, stretched, wrinkled or folded) were selected for morphological analyses; these were fixed in 4% formaldehyde solution and preserved for four days, then the specimens were transferred into 70% ethanol. For molecular analyses, fresh specimens were selected; the testes and clamps were counted and photographed and then the specimens were divided into three pieces, storing the anterior and posterior parts as molecular vouchers. The middle pieces were fixed and preserved in molecular-grade ethanol. Prevalence, expressed as a percentage (infected fish and total number of analysed fish in parentheses), and mean intensity, expressed as the mean with standard deviation, in each host, were calculated according to Bush et al. [7].

### Sequence generation

Ethanol-preserved specimens of *Microcotyle* spp. collected from the four fish species were used for genomic DNA isolation. Total genomic DNA was isolated from the excised pieces of the middle part of the worm body which was dried out at 56 °C before DNA isolation. Chelex™100 Resin (BIO-RAD) was used for extraction (see [8] for details).

Mitochondrial cytochrome *c* oxidase subunit 1 gene (*cox*1, partial fragment) was amplified using primers JB3 (= COI-ASmit1) (forward: 5′-TTT TTT GGG CAT CCT GAG GTT TAT-3′) and JB4.5 (= ASmit2) (reverse: 5′-AAA GAA AGA ACA TAA TGA AAA TG-3′) [9, 10]. Partial fragment (domains D1-D3) of the *28S* rRNA gene was amplified using the primer combination LSU5 (forward: 5′-TAG GTC GAC CCG CTG AAY TTA AGCA-3′) and LSU3′ (reverse: 5′-TAG AAG CTT CCT GAG GGA AAC TTC GG-3′) [11]. Both genes were amplified using puReTaq Ready-To-Go-PCR beads or MiFy^TM^ DNA Polymerase mix (Bioline Inc., Taunton, USA) and PCR amplifications were performed in a total volume of 20 μl containing 8 pmol of each primer and *c*.50 ng of DNA. The thermocycling profiles consisted of: (i) *cox*1: initial denaturation at 94 °C for 5 min, followed by 40 cycles of 92 °C for 30 s, 45.5 °C for 45 s, 72 °C for 90 s, and a final extension step at 72 °C for 10 min; (ii) partial *28S* rDNA: initial denaturation of 94 °C for 4 min, followed by 30 cycles of 94 °C for 1 min, 50 °C for 30 s, 72 °C for 45 s, followed by a final extension step at 72 °C for 7 min.

PCR amplicons were purified using QIAquick TM PCR Purification Kit (Qiagen Ltd., Hilden, Germany). Sequencing reactions were performed using the PCR primers and two additional internal primers in the case of *28S* rRNA gene, i.e. IF15 (forward: 5′-GTC TGT GGC GTA GTG GTA GAC-3′) and IR14 (reverse: 5′-CAT GTT AAA CTC CTT GGT CCG-3′) [12]. Cycle sequencing was carried out at Macrogen Europe Inc. (Amsterdam, the Netherlands).

### Alignment and data analyses

Contiguous sequences were assembled in MEGA v.6 [13] and alignments with currently available sequences for *Microcotyle* spp. in the GenBank database (retrieved on 25th July 2019) were constructed using MAFFT v.7 [14] under default gap parameters on EMBL-EBL bioinformatics web platform (http://www.ebi.ac.uk/Tools/msa/mafft). The outgroup choice was based on previous phylogenies of the group [15–17]. The *cox*1 alignment (381 nt) comprised a total of 12 newly generated sequences and 20 sequences for 10 species available on GenBank. *Bivagina pagrosomi* ex *Sparus aurata* (L.) (GenBank: Z83003) was used as the outgroup. The *28S* alignment (823 nt) comprised 4 newly generated sequences and 10 sequences available on GenBank. *Bivagina pagrosomi* ex *S. aurata* (GenBank: Z83002) was used as the outgroup. Distance matrices (using the uncorrected p-distance model) were calculated in MEGA v. 6. Neighbour-joining analyses based on Kimura 2-parameter distances were also performed in MEGA v.6 with nodal support estimated using 1000 bootstrap resamplings. Model-based Bayesian inference (BI) and maximum likelihood (ML) analyses were carried out using MrBayes v.3.2.6 on XSEDE at the CIPRES Science Gateway v. 3.3 [18] and PhyML v.3.0 [19] as an online execution on the ATGC bioinformatics platform (http://www.atgc-montpelier.fr/) with a non-parametric bootstrap validation of 1000 pseudoreplicates, respectively. The MCMC chains were run for 10,000,000 generations with trees sampled every 1000 generation. Posterior probability and mean marginal likelihood values were calculated. The first 25% of the sampled trees were discarded as ‛burn-inʼ. Prior to analyses, jModelTest v.2.1.4 [20, 21] was used to select the best-fitting models of nucleotide substitution under the Akaikeʼs information criterion. These were the general time-reversible model with gamma distributed among-site rate variation and estimates of invariant sites (GTR+Г+I) for the *cox*1 dataset and the Hasegawa-Kishino-Yano model (HKY) for the *28S* dataset. Consensus topologies and nodal supports were visualized in FigTree v.1.4.3 [22], posterior probabilities (pp) and bootstrap support (bs) values are summarised on the BI trees (as pp/bs).

### Morphological analyses

**Fig. 1:**
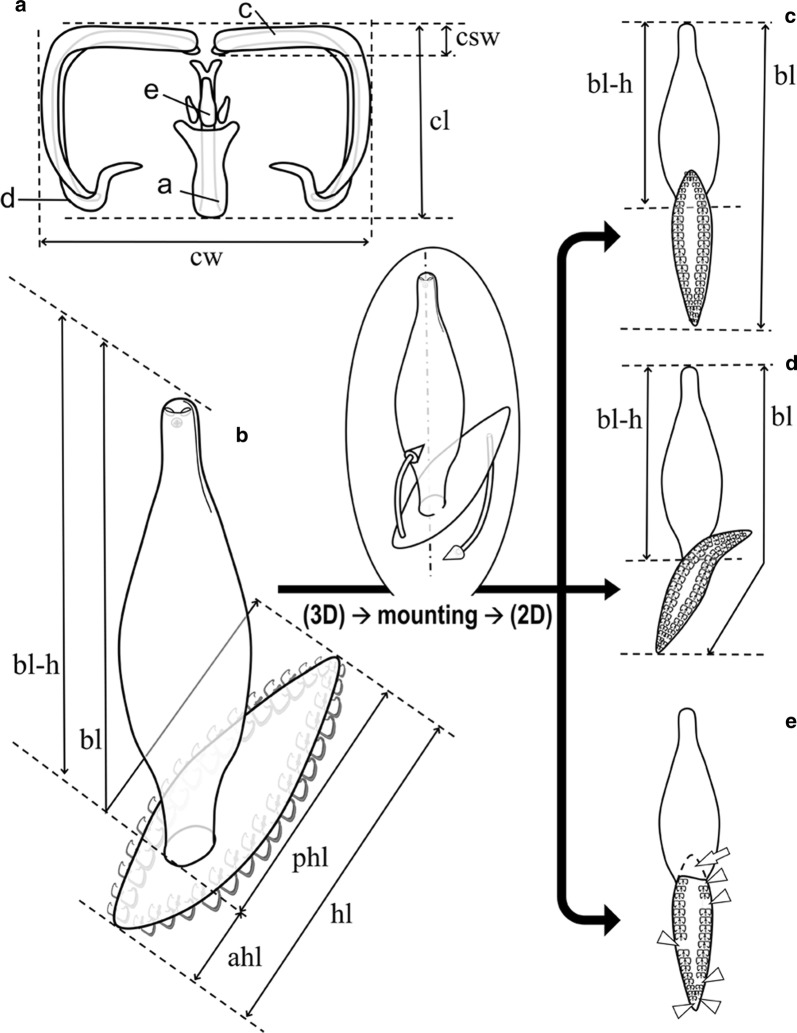
Schematic drawings for measurements of microcotylid clamps and haptors. **a** Clamp measurements: ‘a’, ‘c’, ‘d’ and ‘e’, microcotylid sclerites according to Llewellyn [47]. **b**–**e** Body outlines, number of clamps and measurements in mounted microcotylids: unmounted specimen in 3D view (**b**); mounted specimens in 2D view (**c**–**e**). Haptor anterior lobe lays on the body in **c** and **e**, haptor obliquely mounted in **d** (anterior lobe not laying on the body); **e** represents damaged specimen with missing pieces (arrow) and clamps (arrowheads). *Abbreviations*: ahl, anterior haptor lobe length; bl, body length; bl-h, body length without haptor; cl, clamp length; cw, clamp width; csw, ‘c’ sclerite width; hl, haptor length; phl, posterior haptor lobe length

Parasites selected for morphological analyses were stained with iron acetocarmine, dehydrated through an ethanol series, cleared in dimethyl phthalate and prepared as permanent mounts in Canada balsam. After mounting, there was a second selection of specimens suitable for morphological studies, i.e. only specimens in optimal condition (not broken, contracted, stretched, wrinkled or folded). Parasites were examined using a light microscope Nikon Optiphot-2 (Nikon Instruments, Tokyo, Japan) with differential interference contrast at magnifications of 400–1000×. A total of 86 specimens of *Microcotyle* spp. were selected and drawn (*n* = 22 ex *B. boops*; *n* *=* 21 ex *D. dentex*; *n* *=* 23 ex *P. erythrinus*; *n* *=* 20 ex *Pa. pagrus*). Drawings were made with the aid of a drawing tube attached to a light microscope Nikon Optiphot-2. Measurements were taken from digitalized illustrations using ImageJ v.1.48 software [23] and expressed in micrometers as the range followed by the mean in parentheses unless otherwise stated. When characters were visible, a total of 52 morphometric measurements were taken from each specimen. Clamp thickness was estimated as both the maximum width of the distal end of the antero-lateral sclerite (‘c’, see Fig. 1a) and its relation to the clamp length. The type-specimens were deposited in the Collection of the Natural History Museum (NHMUK), London, UK.

To look for patterns of separation between *Microcotyle* spp. specimens from different host species, a principal components analysis (PCA) was applied to a dataset of 86 specimens using morphometrical variables associated with body shape. Prior to the analysis, the data were divided by total body length to account for the effect of body size while visualising possible morphometric differences between species. The specimens were identified as *M. erythrini* (*n* = 23 ex *P. erythrinus*; *n* = 20 ex *Pa. pagrus*), *M. isyebi* (*n* = 22 ex *B. boops*) and *Microcotyle whittingtoni* n. sp. ex *D. dentex* (*n* = 21).

## Results

### Molecular identification

A total of 12 *cox*1 and four *28S* rDNA sequences were generated for the newly collected specimens of *Microcotyle* spp. from the four fish species from the Western Mediterranean off Spain. Partial *cox*1 (434 nt) sequences were generated for a total of 12 isolates, i.e. 4 *M. isyebi* ex *B*. *boops*, 6 *M. erythrini* (4 ex *Pa*. *pagrus* and 2 ex *P*. *erythrinus*) and 2 *M. whittingtoni* n. sp. ex *D. dentex*. Partial *28S* rDNA sequences (1238–1527 nt) were generated for a representative subset of the specimens used for *cox*1 sequence generation; single sequences per species were used for the reconstruction of the *28S* rDNA phylogeny. The newly generated sequences for the isolates recovered in the present study were analysed in two separate datasets together with all currently available sequences in the GenBank database for *Microcotyle* spp. (see Table 1 for details on the ingroup taxa used in the analyses). Posterior probabilities (pp) and bootstrap support (bs) values are summarised on the BI trees (as pp/bs).

**Table 1:** Summary data for the isolates of *Microcotyle* spp. used in the phylogenetic analyses

Parasite species	Host species	Isolate	FAO Fishing Area	GenBank ID		Source
				*cox*1	*28S*	
*M. algeriensis* Ayadi, Gey, Justine & Tazerouti, 2016	*Scorpaena notata* Rafinesque	MO-01	WM	KX926443		Ayadi et al. [24]
	*Scorpaena notata*	MO-02	WM	KX926444		Ayadi et al. [24]
	*Scorpaena notata*	MO-03	WM	KX926445		Ayadi et al. [24]
*M. archosargi* MacCallum, 1913	*Archosargus rhomboidalis* (L.)	81	WCA		MG586867	Mendoza-Franco et al. [25]
*M. arripis* Sandars, 1945	*Arripis georgianus* (Valenciennes)		SA		GU263830	Catalano et al. [26]
*M. caudata* Goto, 1894	*Sebastes inermis* Cuvier	MC06	NWP	LC472527		Kamio & Ono (unpublished data)
	“*Sebastes inermis* species complex”	MC12	NWP	LC472528		Kamio & Ono (unpublished data)
	“*Sebastes inermis* species complex”	MC18	NWP	LC472529		Kamio & Ono. (unpublished data)
	“*Sebastes inermis* species complex”	MC20	NWP	LC472530		Kamio & Ono (unpublished data)
	“*Sebastes inermis* species complex”	MC24	NWP	LC472531		Kamio & Ono (unpublished data)
*M. erythrini* van Beneden & Hesse, 1863	*Pagellus erythrinus* (L.)	MePe1	WM		**MN814848**	Present study
	*Pagellus erythrinus*	MePe2	WM	**MN816012**		Present study
	*Pagellus erythrinus*	MePe3	WM	**MN816013**		Present study
	*Pagellus erythrinus*		WM	AY009159		Jovelin & Justine [15]
	*Pagellus erythrinus*		WM		AM157221	Badets et al. [12]
	*Pagrus pagrus* (L.)	MePp1	WM	**MN816014**	**MN814849**	Present study
	*Pagrus pagrus*	MePp2	WM	**MN816015**		Present study
	*Pagrus pagrus*	MePp3	WM	**MN816016**		Present study
	*Pagrus pagrus*	MePp4	WM	**MN816017**		Present study
*M. isyebi* Bouguerche, Gey, Justine & Tazerouti, 2019	*Boops boops* (L.)	MiBb1	WM	**MN816018**	**MN814850**	Present study
	*Boops boops*	MiBb2	WM	**MN816019**		Present study
	*Boops boops*	MiBb3	WM	**MN816020**		Present study
	*Boops boops*	MiBb4	WM	**MN816021**		Present study
	*Boops boops*	MO01	WM	MK317922		Bouguerche et al. [3]
*M. sebastis* Goto, 1894	*Sebastes* sp.		NSP		AF382051	Olson & Littlewood [16]
*Microcotyle* sp. AKV-2016	*Nemipterus japonicas* (Bloch)	VII37_12	EAS		KU926692	Verma & Agrawal (unpublished data)
*Microcotyle* sp. DG-2016	*Helicolenus dactylopterus* (Delaroche)	MO-04	WM	KX926446		Ayadi et al. [24]
	*Helicolenus dactylopterus*	MO-06	WM	KX926447		Ayadi et al. [24]
	*Sebastes schlegelii* Hilgendorf		NWP	DQ412044		Park et al. [27]
*Microcotyle* sp. YK-2019	*Sebastiscus marmoratus* (Cuvier)	MK02	NWP	LC472525		Kamio & Ono (unpublished data)
*Microcotyle* sp. YK-2019	*Sebastiscus marmoratus*	MK01	NWP	LC472526		Kamio & Ono (unpublished data)
*Microcotyle* sp. 1 SC-2018	–		NWP		MH700256	Chou (unpublished data)
*Microcotyle* sp. 2 SC-2018	–		NWP		MH700266	Chou (unpublished data)
Microcotylidae sp. M10	*Sebastes* sp.	M10	NWA		EF653385	Aiken et al. [28]
Microcotylidae sp. M11	*Argyrosomus japonicus*	M11	SA		EF653386	Aiken et al. [28]
*M. visa* Bouguerche, Gey, Justine & Tazerouti, 2019	*Pagrus caeruleostictus* (Valenciennes)	PacoerMO01	WM	MK275652		Bouguerche et al. [2]
	*Pagrus caeruleostictus*	PacoerMO02	WM	MK275653		Bouguerche et al. [2]
	*Pagrus caeruleostictus*	PacoerMO03	WM	MK275654		Bouguerche et al. [2]
*M. whittingtoni* n. sp.	*Dentex dentex* (L.)	MwDd1	WM	**MN816010**	**MN814847**	Present study
	*Dentex dentex*	MwDd2	WM	**MN816011**		Present study
“*Paramicrocotyle*” sp. FAS-2014^a^	*Pinguipes chilensis* (Valenciennes)		SWP	KJ794215		Oliva et al. [29]
Outgroup						
*Bivagina pagrosomi* (Murray, 1931)	*Sparus aurata* L.^b^		SWP	Z83003	Z83002	Littlewood et al. [10]

^a^Genus synonymized with *Microcotyle* [2, 30]

^b^As *Sparus auratus* in Littlewood et al. [10]

*Note*: The newly generated sequences are indicated in bold

*Abbreviations*: CPS, Central-South-East Pacific; EAS, Eastern Arabian Sea; NS, North Sea; NWA, North-West Atlantic; NWP, North-West Pacific; SA, Southern Australia; SWP, South-West Pacific; WCA Western-Central Atlantic; WM, Western Mediterranean, –, not specified

**Fig. 2:**
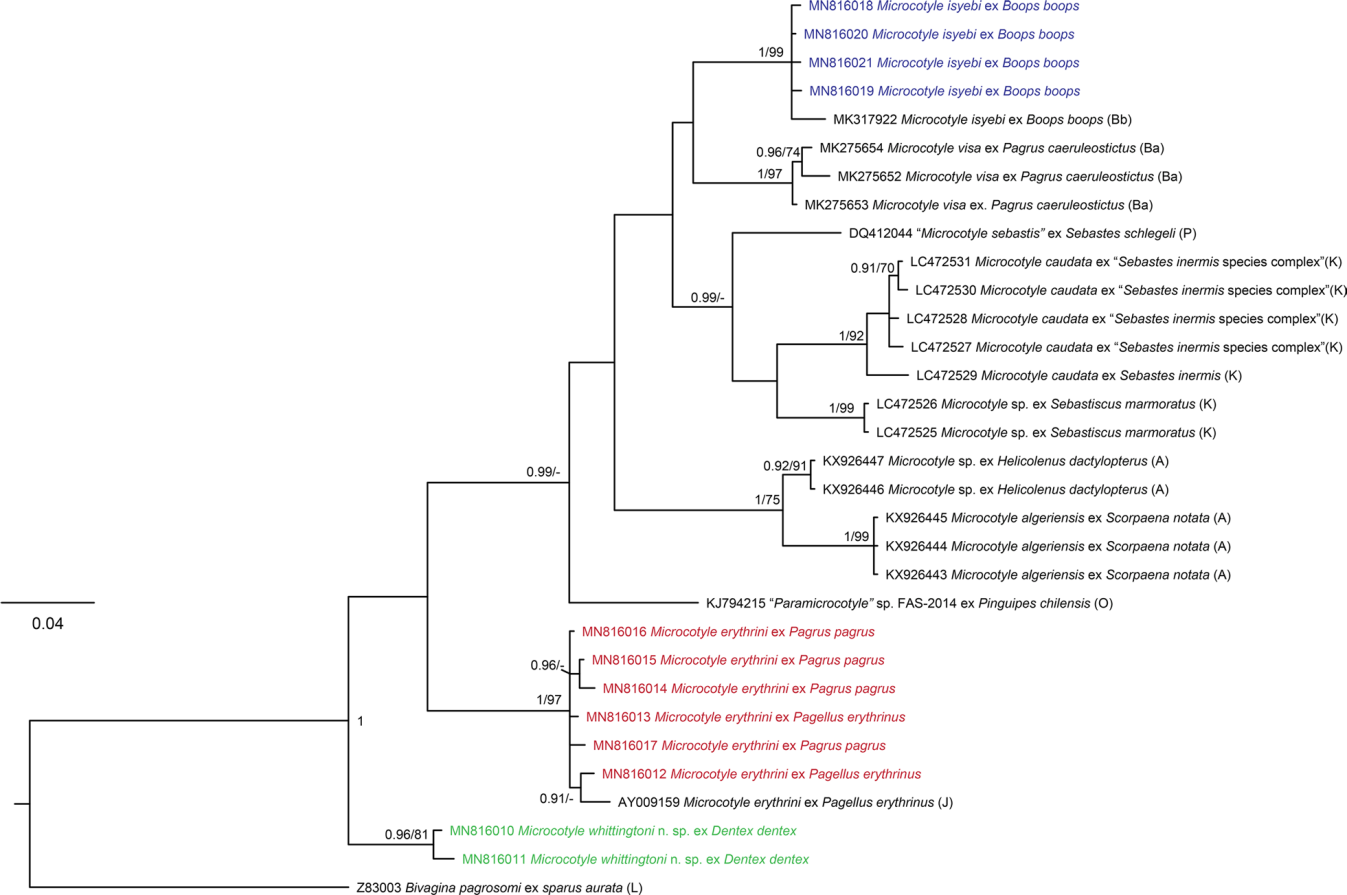
Bayesian inference (BI) phylogram based on the mitochondrial *cox*1 dataset for *Microcotyle* spp. *Bivagina pagrosomi* was used as the outgroup. Posterior probabilities and bootstrap support values are shown at the nodes; only values > 0.90 (BI) and 75% (ML) are shown. The scale-bar indicates the expected number of substitutions per site. Sequence identification is as in GenBank, followed by a letter: A, Ayadi et al. [24]; Ba, Bouguerche et al. [2]; Bb, Bouguerche et al. [3]; J, Jovelin & Justine [15]; K, Kamio & Ono (unpublished); O, Oliva et al. [29]; P, Park et al. [27]

The newly generated *cox*1 sequences were analysed together with 19 published sequences for *Microcotyle* spp. (Table 1). Phylogenetic analysis revealed that the newly sequenced isolates belonged to 3 species: *M*. *erythrini* ex *P*. *erythrinus* and *Pa*. *pagrus*; *M*. *isyebi* ex *B*. *boops*; and *M. whittingtoni* n. sp. ex *D*. *dentex*. The tree from the BI analysis is provided in Fig. 2 together with the statistical support from the ML analysis. The four isolates recovered from *B*. *boops* clustered together with an isolate of *M*. *isyebi* from the same host species reported from the southern coast of the Western Mediterranean off Algeria [3]. The sequences for the isolates recovered from *Pa*. *pagrus* and *P*. *erythrinus* clustered together with the published sequences for *M*. *erythrini* ex *P*. *erythrinus* from the Western Mediterranean off France [15]. The two sequences for *M. whittingtoni* n. sp. ex *D*. *dentex* clustered together in a basal clade to the remaining representatives of *Microcotyle* spp. All of the above clades were strongly supported in both BI and ML analyses. Overall, the *cox*1 phylogeny (Fig. 2) recovered three groups of sister species within the *Microcotyle* although with poor support: (i) *M. isyebi* and *M*. *visa* Bouguerche, Gey, Justine & Tazerouti, 2019; (ii) *M*. *caudata* Goto, 1894 and an unidentified *Microcotyle* sp. ex *Sebastiscus marmoratus* (Cuvier) from the North-West Pacific off Japan; and (iii) *M*. *algeriensis* Ayadi, Gey, Justine & Tazerouti, 2016 ex *Scorpaena notata* Rafinesque and *Microcotyle* sp. ex *Helicolenus dactylopterus* (Delaroche) (syn. *M. sebastis sensu* Radujković & Euzet, (1989) [31]) (both reported from the Western Mediterranean off Algeria). The single sequence for ‘*Microcotyle sebastis*’ was close to the *M*. *caudata*-*Microcotyle* sp. from off Japan, and an isolate originally identified as “*Paramicrocotyle* sp.” (genus synonymised with *Microcotyle* [30]) ex *Pinguipes chilensis* Velenciennes from the South-East Pacific off Chile, was recovered as sister species to the major clade comprising the previously reported representatives from the Mediterranean, North-East Atlantic, Indian Ocean and the North-West Pacific. *Microcotyle erythrini* was recovered apart from the above-mentioned main multi-taxon clade albeit with low nodal support.

The intraspecific sequence divergence (see Additional file 1: Table S1) within the newly generated *cox*1 sequences ranged between 0.2–1.4% (1–6 nt difference) for *M*. *erythrini* (ex *P. erythrinus* and *Pa. pagrus*); 0.2–0.5% (1–2 nt difference) for *M*. *isyebi* and 1.4% (6 nt difference) for *M. whittingtoni* n. sp. ex *D. dentex*. The newly generated sequences for the isolates of *M*. *isyebi* from off Spain differed by 1.4–1.7% (4–5 nt) from *M*. *isyebi* from off Algeria; these for *M*. *erythrini* differed by 2.1–2.8% (6–8 nt) from the published isolate from off Corsica (GenBank: AY009159); and the two isolates of *M. whittingtoni* n. sp. ex *D*. *dentex* differed substantially from both *M*. *isyebi* and *M*. *erythrini*, i.e. by 14.4–15.2% (43–56 nt) and by 10.8–13.5% (41–44 nt), respectively. The overall sequence divergence among the species of *Microcotyle* ranged between 4.5–18.5% (17–62 nt difference).

**Fig. 3:**
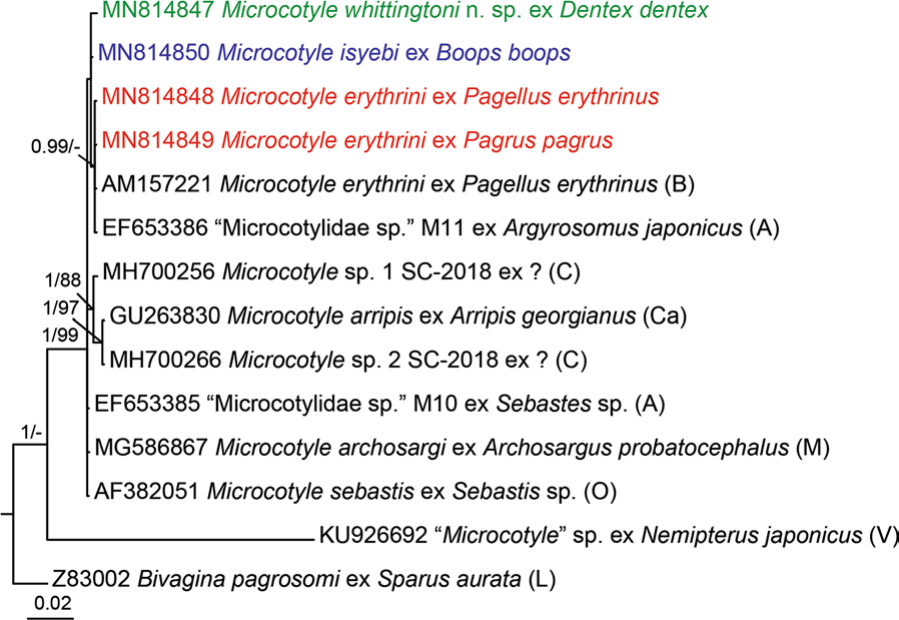
Bayesian inference (BI) phylogram based on the partial *28S* rDNA sequences (domains D1-D3) for *Microcotyle* spp. *Bivagina pagrosomi* was used as the outgroup. Posterior probabilities and bootstrap support values are shown at the nodes; only values > 0.90 (BI) and 75% (ML) are shown. The scale-bar indicates the expected number of substitutions per site. Sequence identification is as in GenBank, followed by a letter: A, Aiken et al. [28]; B, Badets et al. [12]; C, Chou (unpublished data); Ca, Catalano et al. [26]; L, Littlewood et al. [10]; M, Mendoza Franco et al. [25]; O, Olson & Littlewood [16]; V, Verma & Agrawal (unpublished)

Both, ML and BI analyses for the *28S* dataset yielded congruent tree topologies (Fig. 3) and high nodal support for most of the clades. Most of the species of *Microcotyle* clustered in a single multi-taxon clade with the unpublished sequence for an isolate identified as *Microcotyle* sp. ex *Nemipterus japonicus* (Bloch) from the Indian Ocean as a distinct, basal species. The newly generated sequences for *M*. *erythrini* ex *P. erythrinus* and *Pa. pagrus*, clustered in a strongly supported clade together with a previously published sequence for *M*. *erythrini* ex *P. erythrinus* off Corsica, France and a sequence for “Microcotylidae sp.” M11 ex *Argyrosomus japonicus* (Temminck & Schlegel) from off Australia. *Microcotyle whittingtoni* n. sp. and *M*. *isyebi* clustered together as close relatives of *M*. *erythrini* + “Microcotylidae sp.” M11. *Microcotyle arripis* Sandars, 1945 from the South-West Pacific off Australia and an isolate provisionally identified as *Microcotyle* sp. 2 from off China clustered together in a strongly supported subclade.

The novel *28S* sequences for *M*. *erythrini* recovered from the two fish host species differed by a single base. The novel sequence for *M*. *erythrini* from *P*. *erythrinus* was identical with the published sequence ex *P*. *erythrinus* (GenBank: AM157221) in the Western Mediterranean (see Additional file 2: Table S2). The *28S* rDNA sequences for *M*. *isyebi* and *M. whittingtoni* n. sp. differed from the *M*. *erythrini* isolates by 1 and 3 nt (0.1 and 0.4%), respectively, and by 2 nt (0.2%) between themselves. *Microcotyle* sp. AKV-2016 (KU926692) differed substantially from the remaining *Microcotyle* spp., i.e. by 80–116 nt (12.8–14.7%) corresponding to intergeneric-level differences. The overall sequence divergence among the species of *Microcotyle* ranged between 1–8 nt (0.1–1.0%).

### Morphological data

#### ***Microcotyle erythrini*** van Beneden & Hesse 1863 (***sensu stricto***)

***Hosts***: *Pagellus erythrinus* (L.) (type-host), common pandora [weight: 98.9–160.0 g (123 ± 17.3 g); standard length: 15.8–23 cm (17.5 ± 1 cm)] off Guardamar del Segura, Spain; *Pagrus pagrus* (L.), red porgy [weight: 84.2–289.3 g (175.0 ± 44.5 g); standard length: 13.4–20.5 cm (17.12 ± 1.5 cm)], off Guardamar del Segura, Spain (both Perciformes: Sparidae).

***Locality***: Off Guardamar del Segura, Western Mediterranean off Spain. Other localities with valid records: off Brest, France (type-locality); Boka Kotorska Bay and off Montenegro coast, Montenegro; off Sète, France.

***Voucher material***: Specimens from *P. erythrinus* (*n* = 3) and *Pa. pagrus* (*n* = 3) from off Guardamar del Segura are deposited in the Natural History Museum, London, UK (NHMUK.2019.12.10.6-8 and NHMUK.2019.12.10.9-11, respectively); the remaining material from Guardamar del Segura is deposited in the Parasitological Collection of the Cavanilles Institute of Biodiversity and Evolutionary Biology, University of Valencia, Spain.

***Infection parameters***: *P. erythrinus* (*n* *=* 40): prevalence, 51% (21 out of 40); mean intensity, 2.2 ± 3.8; *Pa. pagrus* (*n* *=* 30); prevalence, 45% (14 out of 30); mean intensity, 2.2 ± 1.7.

***Site on host***: Gill filaments.

***Representative DNA sequences***: GenBank accession numbers: MN816012 and MN816013 ex *P. erythrinus*; MN816014, MN816015, MN816016 and MN816017 ex *Pa. pagrus* (*cox*1); MN814848 ex *P. erythrinus* and MN814849 ex *Pa. pagrus* (*28S*).

**Description**

**Fig. 4:**
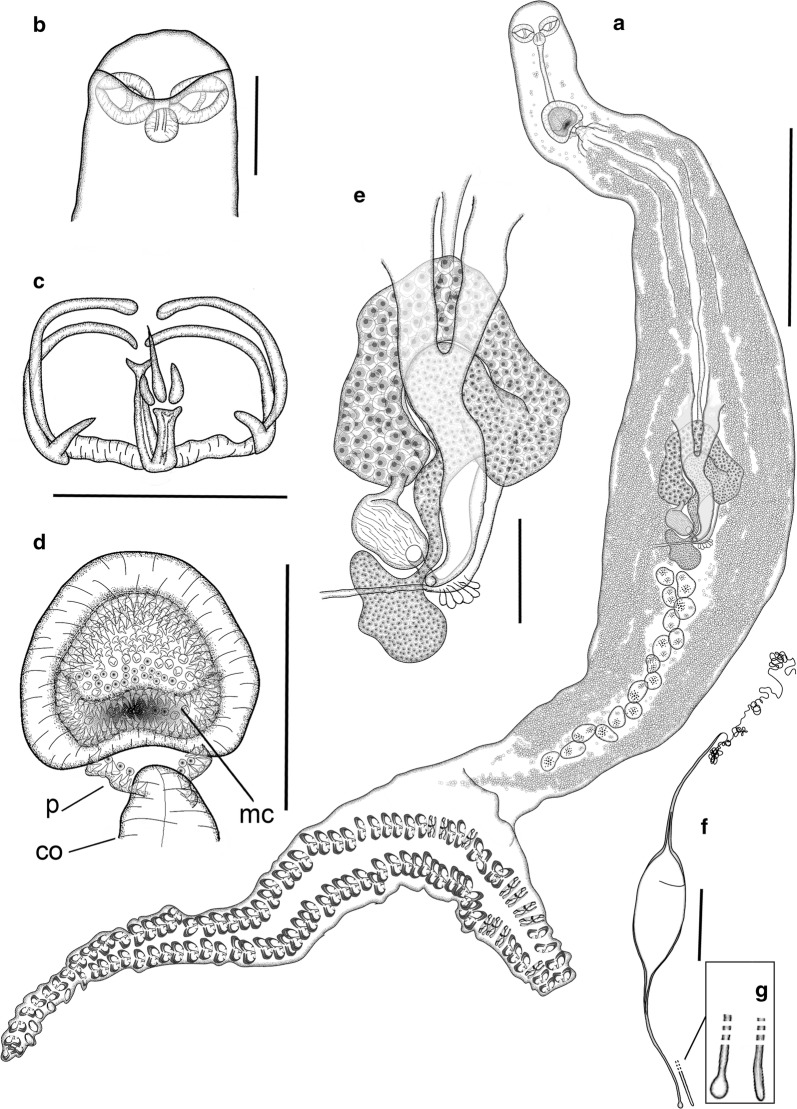
*Microcotyle erythrini* van Beneden & Hesse (1863) (*sensu stricto*) ex *Pagellus erythrinus* (L.) from off Guardamar del Segura, Spain. All drawings from the same voucher specimen. **a** Whole mount. **b** Anterior body end. **c** Clamp. **d** Genital atrium, including copulatory organ. **e** Germarium. **f** Egg. **g** Detail of abopercular egg filament end. *Abbreviations*: co, copulatory organ; mc, main chamber of the genital atrium; p, small posterior chambers (“pockets” *sensu* Mamaev [44]). *Scale-bars*: **a**, 500 µm; **b, d**–**f**, 100 µm; **c**, 50 µm

[Based on 43 mature adults (23 ex *P. erythrinus* and 20 ex *Pa. pagrus*), except where otherwise indicated; data in the description are reported as mean ± SD for specimens ex *P. erythrinus* [mean ± SD ex *Pa. pagrus*]; ranges are provided in Table 2; Fig. 4]. Body fusiform, elongate, slender, 3532 ± 918 [3630 ± 835] long, 182 ± 38 [182 ± 37] wide at level of genital atrium and 249 ± 92 [258 ± 57] wide at level of testes, tapered anteriorly up to 566 ± 107 (*n* *=* 18) [513 ± 141 (*n* *=* 13)] from anterior extremity of body; body laterally narrowed at posterior end of anterior tapered region, 204 ± 55 (*n* *=* 18) [190 ± 39 (*n* *=* 13)] wide, often posteriorly delimited by lateral notches. Haptor dorsoventrally bi-lobed, elongated (haptor length/total body length ratio: 38–62% (44%) [35–60% (42%)]), well differentiated from body, sometimes with peduncle 448 ± 103 (*n* *=* 7) [414 ± 127 (*n* *=* 4)] long, with minimum width 174 ± 36 (*n* *=* 7) [158 ± 47 (*n* *=* 4)]; haptor laterally symmetric, ventrally projected in anterior (ventral) lobe and longer posterior (dorsal) lobe (anterior/posterior haptoral lobe length ratio 60–90% (72%) (*n* *=* 20) [52–81% (66%) (*n* *=* 19)]. Haptor armed with two rows of sessile clamps, 90–124 in number for specimens from both host species, in two lateral frills, joining at anterior and posterior extremities of haptor; with slightly smaller clamps at anterior and posterior margins of haptor. Clamps of “microcotylid” type, slender, “c” sclerite maximum width 3 ± 1 for specimens from both host species and 0.084 ± 0.015 (*n* *=* 25) [0.068 ± 0.019 (*n* *=* 25)] corrected by clamp length; with trident-shaped accessory sclerite (‘e’, see Fig. 1a) formed by thick central bar reaching to distal tips of antero-lateral sclerites ‘c’ and two thin short sclerites directly branched from basis of ‘e’.

**Table 2:** Metrical ranges for *Microcotyle erythrini* (*sensu stricto*), *M. isyebi* and *M. whittingtoni* n. sp. described in this study based on collections from off Guardamar del Segura, Spain, Western Mediterranean

Parasite species	*M. erythrini* (*s.s.*)		*M. isyebi*	*M. whittingtoni* n. sp.
Host species	*P. erythrinus*	*Pa. pagrus*	*B. boops*	*D. dentex*
Sample size	(*n* = 23)	(*n* = 20)	(*n* = 22)	(*n* = 21)
Body length	1998–6215	2042–6183	2355–6401	2719–4569
Body length without haptor	1376–3760	1520–5307	1757–4970	1916–3591
Maximum body width	194–647	189–610	322–966	314–605
Body width at level of buccal suckers	95–179	68–153	95–185	110–173
Body width at level of genital atrium	102–251	109–270	157–322	159–260
Body width at level of testes	156–574	150–357	237–790	225–468
Length of anterior tapered region	359–749	132–687	288–922	457–730
Width of anterior tapered region	135–378	124–242	180–388	147–354
Haptor length	1126–1840	761–1590	702–1436	862–1264
Anterior haptoral lobe length	353–735	307–690	164–339	187–370
Posterior haptoral lobe length	758–1,163	608–991	474–1,226	632–1,038
Peduncle length	339–615	263–574	178–651	199–476
Width of peduncle at connection with haptor	120–246	72–227	92–414	100–369
Minimum peduncle width	120–276	67–227	91–414	81–368
No. of clamps	90–124	90–124	80–110	60–78
Clamp length	20–50	22–42	21–40	22–47
Clamp width	55–86	40–64	46–68	52–75
Sclerite ‘c’ width	2–4	2–3	1–3	3–6
Buccal sucker length	47–80	38–78	50–82	57–90
Buccal sucker width	34–59	19–50	33–63	39–59
Pharynx length	29–47	20–42	29–55	27–41
Pharynx width	23–41	18–34	23–54	25–43
Oesophagus length	159–307	217–343	150–360	209–367
Testes to anterior extremity distance	982–2223	1055–4018	1215–3288	1305–2342
No. of testes	12–20	14–22	19–26	16–27
No. of testis rows	1–2	1–2	2–3	1–3
Testes length	32–115	38–82	32–162	39–112
Testes width	51–116	42–75	52–132	36–88
Testicular area length	298–1007	364–1250	88–301	68–717
Testicular area width	41–193	56–155	408–1558	393–1023
Genital atrium to anterior extremity distance	108–277	206–424	128–355	183–300
Genital atrium length	71–151	71–165	73–177	105–184
Genital atrium width	88–168	54–201	109–255	115–173
No. of spines in the main chamber of the genital atrium	216–408	275–363	253–356	272–391
Length of spines in the main chamber of the genital atrium	4–6	4–6	4–7	4–7
No. of spines in the “pockets” of the genital atrium	20–33	21–41	19–49	34–47
Length of spines in the “pockets” of the genital atrium	6–8	6–9	7–11	7–13
Copulatory organ length	43–100	47–70	52–86	46–107
Copulatory organ width	54–100	33–100	54–118	37–93
Germarium to anterior extremity distance	1093–2149	1381–2460	1141–3240	1240–2240
Vagina to anterior extremity distance	321–550	380–585	328–425	380–550
Germarium length	566–1428	890–1428	896–1792	730–1199
Germarium maximum width	37–127	83–96	47–134	38–86
Seminal receptacle length	144–171	140–185	289–316	182–208
Seminal receptacle width	89–105	80–99	96–112	70–87
Vitellarium to anterior extremity distance	204–560	319–613	244–578	320–566
Length of vitellarium within peduncle	120–450	138–589	18–530	94–621
Length of vitellarium within haptor	10–111	19–42	0–0	17–404
Distance between vitellarium posterior	0–149	0–152	0–462	129–498
Left efferent vitelline duct length	87–287	124–444	127–858	216–467
Right efferent vitelline duct length	114–328	153–377	150–584	202–442
Different vitelline duct length	143–400	224–367	205–456	121–300
Egg length (without filaments)	166–223	145–201	189–230	184–264
Egg width (without filaments)	57–91	57–90	51–88	66–84
Abopercular filament length	95–151	98–147	110–208	40–111

Mouth subventral, within conical vestibule with pair of septate buccal suckers. Pharynx subspherical; oesophagus short; intestinal bifurcation posterior to genital atrium, sometimes at level of atrium. Caeca extend into haptor or peduncle, with inner and intricate external lateral ramifications.

Testes numerous, 12–20 [14–22] in number, dorso-ventrally flattened, subelliptical to irregular, most anterior located at 1536 ± 360 [1922 ± 585] from anterior extremity, post-germarial and pre-haptoral, partially extending into haptor peduncle, arranged in clusters of 1 or 2 rows, with some testes dorso-ventrally overlapped. Vas deferens relatively straight, dorsal to uterus; copulatory organ muscular, 68 ± 22 (*n* *=* 12) [62 ± 7 (*n* *=* 8)], located in posterior part of genital atrium. Genital atrium at 216 ± 40 [267 ± 63] from anterior extremity of body, with wide medial muscular chamber, armed with small conical spines, 216–408 [275–363] in number, communicated with 2 lateral posterior small chambers (“pockets” *sensu* Mamaev, 1989 [44]) armed with longer spines, 20–33 [21–41] in number.

Germarium at 1503 ± 292 (*n* *=* 8) [1758 ± 268 (*n* *=* 6)] from anterior extremity of body; 867 ± 251 (*n* *=* 8) [913 ± 150 (*n* *=* 6)] long, question mark-shaped, with proximal globular germinal area, 71 ± 25 × 110 ± 20 (*n* *=* 8) [76 ± 9 × 121 ± 43 (*n* *=* 6)], connected with narrow straight section, 278 ± 104 × 35 ± 20 (*n* *=*  8) [292 ± 92 × 45 ± 14 (*n* *=* 6)], widening in long distal globular region, 599 ± 50 (*n* *=* 8) [621 ± 51 (*n* *=* 6)] long, with proximal arched section directed dextro-sinistrally, connected to wide arched section directed sinistro-dextrally; maximum width at distal section, 71 ± 28 (*n* *=*  8) [90 ± 10 (*n* *=* 6)]. Oviduct slightly sinuous, including elongated seminal receptacle, 99 ± 7 × 160 ± 12 (*n* *=*  4) [93 ± 8.3 × 163 ± 13 (*n* *=* 2)] directed postero-sinistrally; ending in oötype; Mehlis’ gland well developed.

Vaginal pore mid-dorsal, often imperceptible, at 465 ± 64 (*n* *=* 11) [473 ± 59 (*n* *=* 8)] from anterior extremity. Vitelline follicles dispersed, starting at 356 ± 81 [437 ± 82] from anterior body extremity, in 2 lateral fields surrounding caecal ramifications; vitelline follicles extending within haptor or peduncle in all specimens. Posterior extremities of vitelline fields asymmetrical in 52% [95%] of specimens, distance between fields usually short, 83 ± 54 [89 ± 46]; right field longer in 56% [61%] of specimens with asymmetrical fields; posterior extremities of vitelline fields often joined (83% [45%] of specimens with symmetrical fields). Vitelline ducts Y-shaped (Fig. 4e), with 2 separate efferent ducts, right 204 ± 67 [224 ± 91] long, left 178 ± 77 [304 ± 119] long, joining in common different duct 214 ± 79 [289 ± 67] long, ventral, at germarium level. Eggs fusiform (Fig. 4f), with 2 filaments; opercular filament long, thin, slightly thickened at posterior end; abopercular filament shorter with solid tip, capitate or pointed (Fig. 4g). Opercular end of egg narrowed to connect abruptly with tubular hollow section (1/3–1/7 of total egg length, not including filaments, for specimens for both host species) leading to opercular filament.

**Remarks**

*Microcotyle erythrini* was described by van Beneden & Hesse [4] and mostly characterized by its specific host, *P. erythrinus*, as authors provided limited morphological information (mostly at the generic level) and with no supporting drawing. Parona & Perugia [5] redescribed this species; however the description is unreliable as these authors provided pooled morphological information from material ex *B. boops*, host of *M. isyebi* (see [3] and present study) and ex *P. acarne*, a host not confirmed for *M. erythrini*. Morphological data with pooled information form specimens collected from more than one host species or parasites collected in fish species different from the type-host or other confirmed hosts should not be considered as suitable (see also Additional file 3: Table S3). Several new geographical records of *M. erythrini* ex *P. erythrinus*, exclusively, have been published by other authors since 1863 (see Table 1 in [3]). Among these records, only Radujković & Euzet [31] and Bouguerche et al. [3] provided morphological and morphometric data for specimens off Montenegro and Séte, respectively (see also Additional file 3: Table S3). Here, we provide metrical data (Table 2) for newly collected specimens ex *P. erythrini* and *Pa. pagrus* (new host record) from the Spanish Western Mediterranean. Specimens from these two hosts collected in the present study are genetically and morphologically indistinguishable.

Only considering the specimens reported ex *P. erythrinus* and *Pa. pagrus* by van Beneden & Hesse [4], Radujković & Euzet [31], Bouguerche et al. [3] and the present study [from here onwards *M. erythrini* (*sensu stricto*)], the diagnostic characters of *M. erythrini* (*s.s.*) agree and measurements mostly overlap but wide ranges for some features are still observed (see also Additional file 3: Table S3), which hampers the differentiation from other congeneric species. Paying attention to the characters traditionally used in the taxonomy of *Microcotyle*, the number of clamps (82–132) and the number of testes (9–24) of *M. erythrini* (*s.s.*), combining the information from all descriptions in confirmed hosts (see Table 2 and Additional file 3: Table S3), resemble or overlap with those of several species reported in the Mediterranean (*M. donavini* van Beneden & Hesse, 1863 and *M. pomatomi* Goto, 1899) and in other sparid hosts (*M. isyebi* and *M. visa*). Regarding the traits more recently used to differentiate the species of *Microcotyle*, such as the genital atrium armature and combining the information from all descriptions in confirmed hosts (see Table 2 and Additional file 3: Table S3), *M. erythrini* (*s.s.*) resembles other species with large number of spines in the main chamber (201–408) and “pockets” of the genital atrium (20–41), overlapping with *M. isyebi*, *M. pomatomi*, *M. visa*, *M. whittingtoni *n. sp. and *Microcotyle* sp. ex *H. dactylopterus* (see [24, 31]; numbers estimated from the drawing for *M. pomatomi*) (see Additional file 3: Table S3).

According to the combination of the characters listed above, *M. isyebi*, *M. pomatomi* and *M. visa* appear most similar morphologically to *M. erythrini* (*s.s.*). *Microcotyle pomatomi*, the only species described and reported from a non-sparid host (*Pomatomus saltatrix* (L.); Pomatomidae), is difficult to differentiate due to the numerous circumglobal records and descriptions which have increased abnormally the ranges for the metrical data of this species. Moreover, the only Mediterranean description of *M. pomatomi* (off Turkey, Sezen & Price, 1967 in [32]) is particularly similar to *M. erythrini* (*s.s.*). Detailed morphological and molecular studies are needed to differentiate the two species. The other two species, both sparid parasites, were described as hardly morphologically distinguishable from *M. erythrini*. *Microcotyle visa* was differentiated from *M. erythrini* by the smaller clamp size, larger pharynx and greater number of testes; however, these differences are not completely sufficient to differentiate species as all they overlap (even with those of *M. erythrini* (*s.s.*)) [2]. No diagnostic morphological differences were provided by Bouguerche et al. [3] to distinguish *M. isyebi* from *M. erythrini*, other than body size, different hosts and large genetic divergence based on *cox*1 data. New evidence reported in the present study allows characterizing *M. erythrini* (*s.s.*) based on the size and shape of the haptor which is relatively longer in relation to body length (35–62% *vs* 27–34% in *M. visa* and 21–32% in *M. isyebi*) and the greater ratio of anterior/posterior haptoral lobe length (52–90% *vs* 34–50% in *M. visa* and 17–52% in *M. isyebi*) (data for *M. visa* estimated from figure 3A in Bouguerche et al. [2]; those for *M. isyebi* from the present study). The anterior/posterior haptoral lobe length ratio range for *M. erythrini* (*s.s.*) is very close to the upper range limits for these two species; however, the ratio was > 60% in some of the *M. erythrini* (*s.s.*) specimens examined here (11 out of 21 ex *P. erythrinus* and 5 out 18 ex *Pa. pagrus*). Additionally, vitelline fields always extend within the haptor in *M. erythrini* (*s.s.*). Bougherche et al. [3] reported that the left caecum-vitellarium branch of *M. isyebi* extends into haptor; however, both vitellarium fields of the *M. isyebi* specimens analysed in the present study are always prehaptoral. Finally, in the new material from the Spanish Mediterranean, the tips of the abopercular filaments of the eggs are solid (capitated or pointed) in *M. erythrini* (*s.s.*) *vs* half cup-shaped to bifid in *M. isyebi*. No information on this trait is available for *M. visa*.

#### ***Microcotyle isyebi*** Bouguerche, Gey, Justine & Tazerouti, 2019

***Host***: *Boops boops* (L.) (type-host) (Teleostei: Sparidae), bogue [weight: 112.9–216.7 g (157.2 ± 22.3 g); standard length: 19.8–24.0 cm (21.7 ± 1 cm)], off Guardamar del Segura, Spain.

***Locality***: Off Guardamar del Segura, Western Mediterranean off Spain. Other localities with valid records: off Bouharoun, Algeria (type-locality) and off Granada, Spain.

***Voucher material***: Three specimens from off Guardamar del Segura are deposited in the Natural History Museum, London, UK (NHMUK.2019.12.10.12-14); the remaining material from Guardamar del Segura is deposited in the Parasitological Collection of the Cavanilles Institute of Biodiversity and Evolutionary Biology, University of Valencia, Spain.

***Infection parameters***: Prevalence: 70% (28 out of 40); mean intensity, 4.96 ± 4.46 (*n* *=* 40).

***Site on host***: Gill filaments.

***Representative DNA sequences***: GenBank accession numbers: MN816018, MN816019, MN816020 and MN816021 (*cox*1); MN814850 (*28S*).

**Description**

**Fig. 5:**
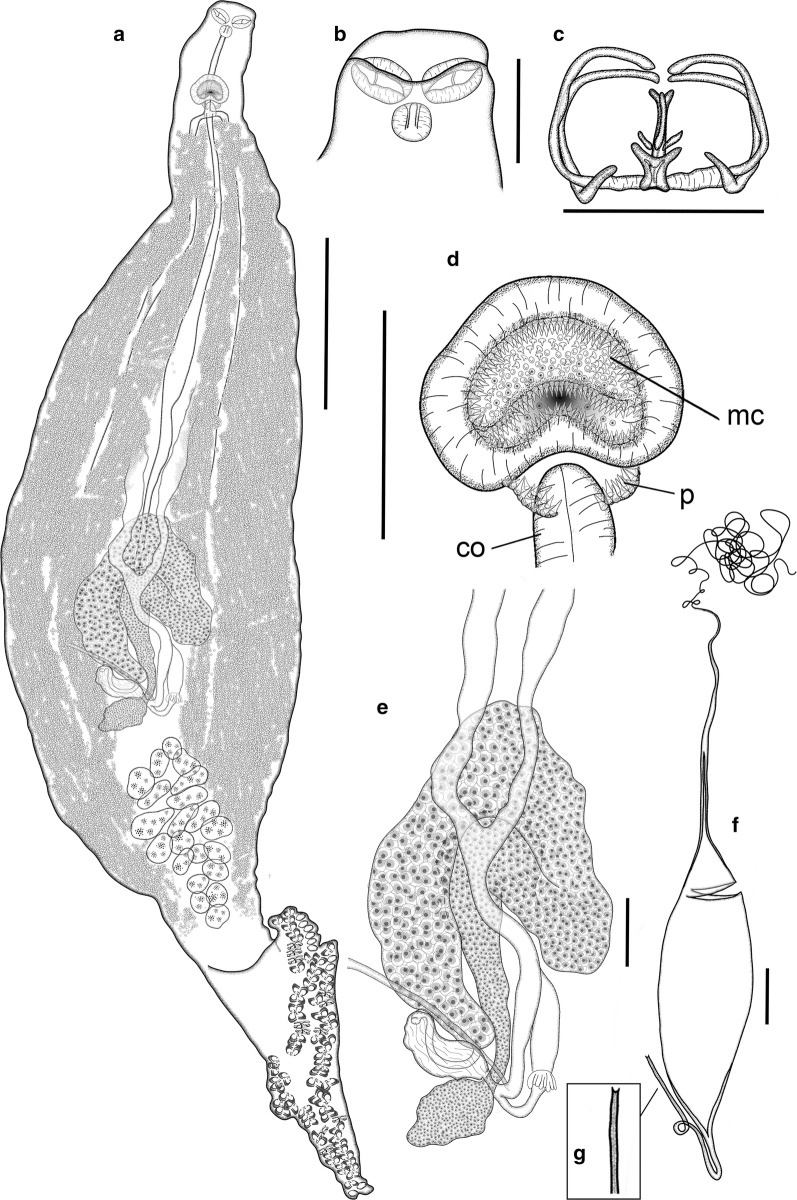
*Microcotyle isyebi* Bouguerche, Gey, Justine & Tazerouti, 2019 ex *Boops boops* (L.) from off Guardamar del Segura, Spain. All drawings are from the same voucher specimen, except for the egg. **a** Whole mount. **b** Anterior end. **c** Clamp. **d** Genital atrium, including copulatory organ. **e** Germarium. **f** Egg. **g** Detail of abopercular egg filament. *Abbreviations*: co, copulatory organ; mc, main chamber of the genital atrium; p, small posterior chambers (“pockets” *sensu* Mamaev [44]). *Scale-bars*: **a**, 500 µm; **b**, **d**–**f**, 100 µm; **c** 50 µm

[Based on 22 mature adults (paragenophores *sensu* [3]); data in the description are reported as mean ± SD, ranges are provided in Table 2; Fig. 5]. Body fusiform, stout to elongate. Anterior region tapered, 585 ± 147 long, posteriorly delimited by lateral notches, which narrow body to 271 ± 57 wide. Body width 221.0 ± 45 at level of genital atrium, 411 ± 130 at level of testes. Haptor relatively short [haptor length/total body length ratio 21–32% (26%)], dorsoventrally bi-lobed, well differentiated from body by peduncle 402 ± 149 long, with minimum width 238 ± 72 (*n* *=* 19); laterally symmetric, divided into anteriorly projected very short ventral lobe and longer posterior lobe (dorsal) [anterior haptoral lobe/posterior haptoral lobe length ratio 17–52% (33%)]. Haptor armed with two rows of sessile clamps, 80–110 in number, in two lateral frills, joining at anterior and posterior extremities of haptor. Clamps at anterior and posterior extremities of haptor slightly smaller. Clamps of “microcotylid” type, slender, “c” sclerite maximum width 2 ± 1 and 0.057 ± 0.021, corrected by clamp length (*n* *=* 25); with trident-shaped accessory sclerite (‘e’, see Fig. 1) formed by long thick central bar reaching to distal tips of antero-lateral sclerites ‘c’ and 2 thin branches directly ramified from basis of ‘e’.

Mouth subventral, within funnel-shaped vestibule with pair of septate buccal suckers. Pharynx subspherical; oesophagus short, connected to intestinal bifurcation at posterior margin of genital atrium, or more posterior. Caeca extend up to haptor peduncle, with inner and external lateral ramifications (external more profuse).

Testes numerous, 19–26 in number, dorso-ventrally flattened, sub-elliptical to irregular, grouped in testicular fields, most anterior located at 2016 ± 476 from anterior extremity of body, post-germarial and pre-haptoral (partially extending into haptor peduncle); testes arranged in clusters of 2 or 3 rows, with some testes overlapped dorso-ventrally. Vas deferens wide, straight, dorsal to uterus, terminating in short muscular copulatory organ, 67 ± 13 × 81 ± 21 (*n* *=* 10), located in posterior part of genital atrium. Genital atrium at 233 ± 45 from anterior extremity of body, formed by main wide medial muscular chamber, armed with small conical spines, 253–356 in number, followed by 2 small chambers (“pockets”) located posteriorly, at both sides of copulatory organ; “pockets” armed with conical spines, 19–49 in number, longer than spines in main chamber (see Table 2).

Germarium 1274.7 ± 199 long (*n* *=* 10), question mark-shaped, at 1674 ± 472 (*n* *=* 10) from anterior extremity of body; proximal globular germinal area 75 ± 28 × 135 ± 38 (*n* *=* 10), connected to straight, narrow section 398 ± 104 × 43 ± 14 (*n* *=* 10), widening in tubular region 877.1 ± 231 long (*n* *=* 10) with proximal arched dextro-sinistral section, connected to wider distal arch directed sinistro-dextrally, with maximum distal width 83 ± 28 (*n* *=* 10). Oviduct directed postero-sinistrally ending in oötype, sinistral to germarium, with short sinuous proximal section connected with wide elongated chamber filled with sperm (oviducal seminal receptacle 106 ± 7 × 304 ± 112 (*n* *=* 3). Mehlis’ gland well developed.

Vaginal pore medial, dorsal, unarmed, often unobserved, at 372.8 ± 62 (*n* *=* 8) from anterior extremity. Vitelline follicles dispersed, extending from 391 ± 82 from anterior extremity of body, extended in 2 lateral fields together with caeca and surrounding testes, usually pre-haptoral but partly extending within peduncle. Posterior extremities of vitelline fields mostly different in length (79% of specimens with asymmetrical fields), always unjoined; distance between fields, 0–462, right field longer in 70% of the specimens. Vitelline ducts Y-shaped, with 2 unjoined ducts 339 ± 150 and 357 ± 213 long (right and left, respectively) (*n* *=* 10), joining ventral to germarium in slightly sinuous defferent duct, 337 ± 84 (*n* *=* 10) long. Egg fusiform (Fig. 5f), with 2 filaments; opercular filament long, thin, often with thickened final tip; abopercular filament short, ending in thickened tip, half cup-shaped to bifid (Fig. 5g). Opercular extremity of egg narrowing abruptly in tubular hollow section (1/3–1/6 of total egg length, not including filaments) leading to opercular filament.

**Remarks**

Both morphological and molecular data reported in the present paper agree with the original description of *M. isyebi* based on material from *B. boops* off Algeria [3] and from the Spanish Mediterranean [33]. Parona & Perugia [5] and Akmirza [6] also provided morphological data from specimens identified as *M. erythrini* ex *B. boops* but these were not considered as species diagnostic in the present study as they represent pooled information for parasites ex *B. boops* and another host, *P. acarne*; *Microcotyle* spp. in sparids are highly host species-specific (see [2, 3] and the present study). In the present study, no specimens of *Microcotyle* spp. were found in *P. acarne*.

Some comments on the original diagnosis of the species can be added in light of the data from the description of López-Román & Guevara Pozo [33] and the present study. The range for the number of clamps seems too wide in the original description of *M. isyebi* based on material collected off Algeria (54–102) compared with that reported by López-Román & Guevara Pozo [33] (90–100) and the present study (80–110) based on material collected off Spain. These numbers should be re-examined, especially because this trait is particularly differential among the species of *Microcotyle* and, as previously reported, it could help differentiate *M. isyebi* from other similar species such as *Microcotyle* sp. ex *H. dactylopterus* [24] and *M. whittingtoni* n. sp. (see [3] and Remarks to the new species below). Particular attention must be paid to the lower range for clamp number as a small number of clamps is often related to young or damaged specimens. The number of spines in the main chamber of the genital atrium of *M. isyebi* is also clearly lower in the original description than in the present material (136–230 *vs* 253–356) (see [3] and Table 2), thus enlarging the range for *M. isyebi* and making this trait almost useless in characterizing this species as it overlaps with most of the species except for *M. donavini* and *M. omanae* Machkewskyi, Dimitrieva, Al-Jufaili & Al-Mazrooei, 2013 (with lower and higher number of spines respectively, see Additional file 4: Table S4). The presence of posterior small chambers of the genital atrium (“pockets”) was also reported as diagnostic in the original description of *M. isyebi*; however, this feature requires a further comment. According to Bouguerche et al. [3], “pockets” are absent in *M. archosargi*, *M. lichiae* Ariola, 1899 and *M. pomatomi*; however, this difference seems to be valid only for *M. lichiae*, as these small chambers exist in *M. archosargi* and *M. pomatomi* according to the drawings in [25] and [32], respectively.

In the original description of the species, *M. isyebi* was differentiated from *M. pomatomi* and from *Microcotyle* sp. ex *H. dactylopterus* [24] by traits with overlapping ranges (the number of clamps and spines of the genital atrium for *Microcotyle* sp. ex *H. dactylopterus*) or almost overlapping ranges (the number of clamps and testes for *M. pomatomi*). *Microcotyle pomatomi* and *Microcotyle* sp. ex *H. dactylopterus* [24] require further taxonomic research; *M. pomatomi* has numerous descriptions and synonyms worldwide which have expanded extremely the ranges for morphological features (see [32]; also the only Mediterranean record by Sezen & Price (1967) in [32]), and the morphology *Microcotyle* sp. ex *H. dactylopterus* has been only briefly described [24, 31].

Bouguerche et al. [3] reported that *M. isyebi* is almost indistinguishable from *M. erythrini*. As mentioned above, examination of mature, entire, uncontracted, unstretched and unfolded specimens of this species would be helpful to define or shorten some of the descriptive morphological ranges. Other morphological traits suggested in the present study reveal additional differences. Thus, *M. isyebi* differs from *M. erythrini* (*s.s.*) in having a shorter haptor in relation to body length (21–32 *vs* 35–62%) and a shorter anterior haptoral lobe in relation to posterior haptoral lobe length (17–52 *vs* 52–90%) and from *M. whittingtoni* n. sp. in the possession of slender clamps (ratio “c” sclerite maximum width/total clamp length, 0.027–0.88 *vs* 0.100–0.146; see the Remarks for *M. whittingtoni* n. sp. below).

#### *Microcotyle whittingtoni* n. sp.

*Synonym*: *Microcotyle erythrini* van Beneden & Hesse, 1863 of González González (2005) [36].

***Type-host***: *Dentex dentex* (L.) (Teleostei: Sparidae), common dentex [weight: 204.0–296.2 g (227.5 ± 24 g); standard length: 22.3–20.0 cm (20.8 ± 0.7 cm)], off Guardamar del Segura, Spain).

***Type-locality***: Off Guardamar del Segura, Western Mediterranean off Spain. Other locality with a valid record: off Balearic Islands, Spain.

***Type-material***: The holotype (NHMUK.2019.12.10.1) and 3 paratypes (NHMUK.2019.12.10.2-5) from off Guardamar del Segura are deposited in the Natural History Museum, London, UK; the remaining material from off Guardamar del Segura is deposited in the Parasitological Collection of the Cavanilles Institute of Biodiversity and Evolutionary Biology, University of Valencia, Spain.

***Infection parameters***: Prevalence, 58% (23 out of 40); mean intensity, 4.36 ± 5.18 (*n* *=* 40).

***Site on host:*** Gill filaments.

***Representative DNA sequences***: GenBank accession numbers: MN816010 and MN816011 (*cox*1); MN814847 (*28S*).

***ZooBank registration***: To comply with the regulations set out in article 8.5 of the emended 2012 version of the International Code of Zoological Nomenclature (ICZN, 2012) details of the new species have been submitted to ZooBank. The life Science Identifer (LSID) for *Microcotyle whittingtoni* n. sp. is urn:lsid:zoobank.org:act:5E369A8A-0EA2-4ED2-A3C6-0D6E4CC5A390.

***Etymology***: The new species is named in honour of the late Dr Ian David Whittington, eminent researcher on monogenean biology and taxonomy. His comprehensive, meticulous and brilliant studies have inspired and encouraged fish parasitologists worldwide.

**Description**

**Fig. 6:**
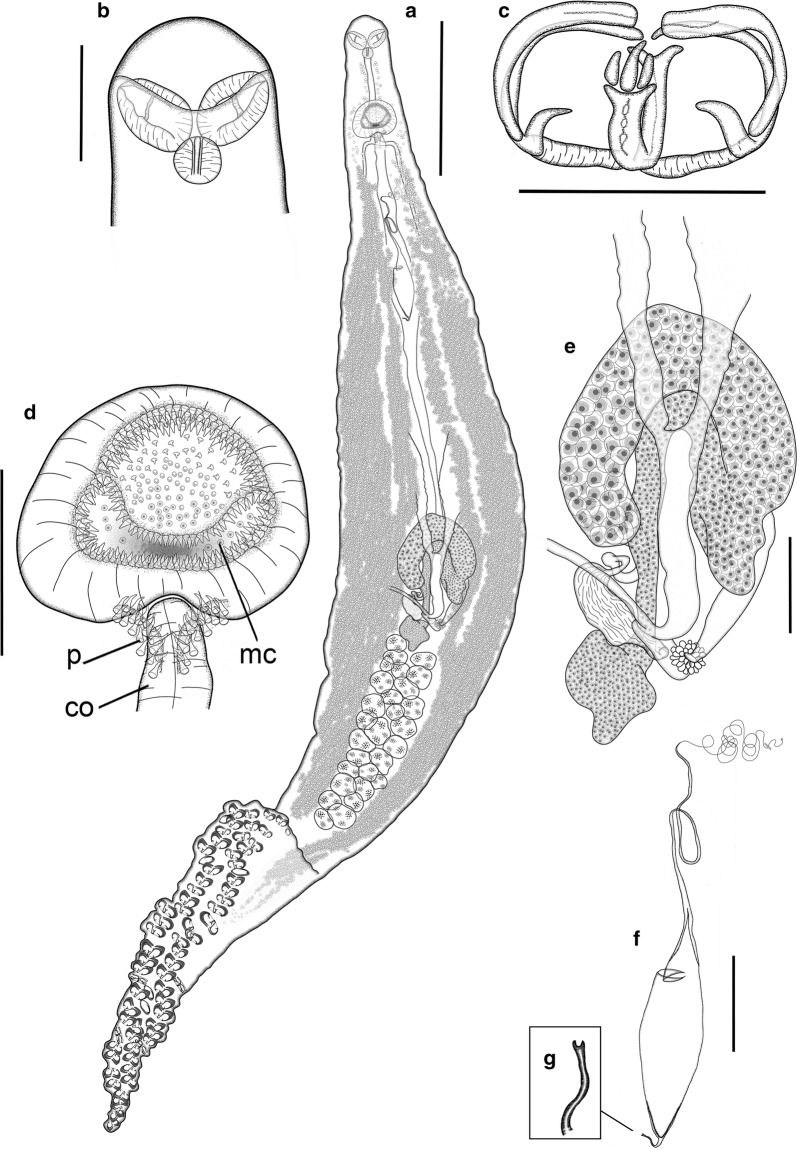
*Microcotyle whittingtoni* n. sp. ex *Dentex dentex* (L.) from off Guardamar del Segura, Spain. Holotype. **a** Whole mount. **b** Anterior end. **c** Clamp. **d** Genital atrium, including copulatory organ. **e** Germarium. **f** Egg. **g** Detail of abopercular egg filament. *Abbreviations*: co, copulatory organ; mc, main chamber of the genital atrium; p, small posterior chambers (“pockets” *sensu* Mamaev [44]). *Scale-bars*: **a**, 500 µm; **b**, **d**–**f**, 100 µm; **c**, 50 µm

[Based on 21 mature adults, except when otherwise indicated; data in the description are reported as mean ± SD; ranges are provided in Table 2; Fig. 6]. Body fusiform, elongate, occasionally slender, 3509 ± 507 long, tapered anteriorly at 563 ± 79 (*n* *=* 20) from anterior extremity of body; anterior tapered region posteriorly delimited by lateral notches, which narrow body to 253 ± 54 (*n* *=* 20) wide. Body 212 ± 27 wide at level of genital atrium and 316 ± 69 wide at testes level. Haptor dorsoventrally bi-lobed, relatively long [haptor length/total body length ratio 24–35% (30%)], well differentiated, sometimes with peduncle [peduncle 309 ± 104 long, with minimum width 204 ± 77 (*n* *=* 6)]; laterally symmetric, with short ventral lobe projected anteriorly and longer posterior (dorsal) lobe [anterior/posterior haptoral lobe length ratio 21–52% (36%)]. Haptor armed with sessile clamps, 60–78 in number, in two rows in lateral frills, joining at anterior and posterior extremities of haptor; clamps slightly smaller at anterior and posterior extremities of haptor. Clamps robust, “c” sclerite maximum width 5 ± 1 and 0.120 ± 0.017 corrected by clamp length (*n* *=* 25); “microcotylid” type with trident-shaped accessory sclerite (‘e’, see Fig. 1) with long thick central bar reaching to distal tips of antero-lateral sclerites ‘c’ and 2 delicate branches ramified from basis of ‘e’.

Mouth subterminal, ventral, with 2 septate buccal suckers within funnel-shaped vestibule; oesophagus short; intestinal bifurcation at level of posterior margin of genital atrium or just proterior. Caeca with inner and profuse external lateral ramifications extending into haptor.

Testes numerous, 16–27 in number, dorso-ventrally flattened, subelliptical to irregular, arranged in clusters of 1–3 rows, with some testes overlapping dorso-ventrally; testicular field at 1807 ± 304 from anterior extremity of body, post-germarial and pre-haptoral, partially extending into haptor peduncle. Vas deferens wide, coursing dorsal to uterus, straight up to short muscular copulatory organ, 80 ± 17 × 60 ± 19 (*n* *=* 10), opening into posterior part of genital atrium. Genital atrium at 240 ± 34 from anterior extremity of body, with muscular wall, formed by wide main medial chamber, covered with tiny conical spines (272–391 in number), connected with 2 postero-lateral small chambers (“pockets”) armed with longer curved spines (34–47 in number), flanking copulatory organ.

Germarium elongated 953 ± 331 long (*n* *=* 11), question mark-shaped, at 1336 ± 167 (*n* *=* 11) from anterior extremity of body; proximal globular germinal area 58 ± 13 × 119 ± 44 (*n* *=* 11) followed by straight narrow section 311 ± 59 × 37 ± 8 (*n* *=* 11), connected with wide tubular region 642 ± 180 (*n* *=* 11) formed by 2 arches crossing first dextro-sinistrally and then sinistro-dextrally, gradually widening up to maximum width of 60 ± 16 (*n* *=* 11) at distal section. Oviduct directed postero-sinistrally ending in oötype; connected to elongated oviducal seminal receptacle 83 ± 7 × 197 ± 12 (*n* *=* 3) by sinuous narrow section. Mehlis’ gland well developed.

Vaginal pore mid-dorsal, unarmed, inconspicuous, at 487 ± 59 from anterior extremity of body (*n* *=* 10). Vitelline follicles small, scattered from 423 ± 59 from anterior extremity of haptor, with lateral fields accompanying caecal ramifications and surrounding testes; vitellarium spread along peduncle and within haptor in all specimens. Posterior extremities of vitelline fields unjoined, always different in length (distance between fields, 308 ± 113; right field longer in 86% of the individuals). Vitelline ducts Y-shaped; efferent ducts 316 ± 81 and 331 ± 79 (right and left, respectively) long (*n* *=* 10) separated up to germarium and ventrally joined in slightly sinuous defferent duct, 228 ± 55 long (*n* *=* 10). Eggs fusiform (Fig. 6f), with 2 filaments; opercular filament very long, thin, with slightly thicker final tip; abopercular filament shorter, ending in half cup-shaped to bifid tip (Fig. 6g). Opercular end of egg narrowing gradually to connect through conical hollow section (1/6–1/8 of total egg length, not including filaments) leading to opercular filament.

**Remarks**

*Microcotyle whittingtoni* n. sp. differs from *M. erythrini* (*s.s.*) by the number of clamps (60–78 *vs* 82–132), the haptor length/total body length ratio (24–35 *vs* 35–62%), the anterior/posterior haptoral lobe length ratio (21–52 *vs* 52–90%) and the shape of the tip of the abopercular egg filament (half cup-shaped to bifid *vs* capitate or pointed). The number of clamps of *M. whittingtoni* n. sp. is particularly low (60–78), similar to other species reported in non-sparid hosts (*M. lichiae* and *Microcotyle* sp. ex *H. dactylopterus* [24]). The ranges for this trait also overlap with those in the original descriptions of two species described from sparids: *M. isyebi* ex *B. boops* (54–102 clamps; see [3]) and *M. visa* ex *Pa. caeruleosticus* (59–126 clamps; see [2]). These ranges are abnormally wide and should be reviewed (see also the Remarks for *M. isyebi* above). The number of testes of *M. whittingtoni* n. sp. (16–27) is a less defining character as the range overlaps the ranges for most *Microcotyle* spp. (see e.g. [34] and Additional file 3: Table S3). Considering the species reported in the Mediterranean or in sparid hosts, this trait is only useful for differentiating the new species from *M. omanae* (34–55 testes) and from *Microcotyle* sp. ex *H. dactylopterus* [24], a species with lower but slightly overlapping number of testes (10–17). Regarding the genital atrium armature, the number of spines in the main chamber (272–391) in the new species overlaps with the ranges for *M. erythrini* (*s.s.*), *M. isyebi* ([3]; present study), *M. pomatomi* and *Microcotyle* sp. ex *H. dactylopterus* [24]. The spines in the “pockets” of the genital atrium in *M. whittingtoni* n. sp. appear to be longer and more curved than those of the other species examined in the present study (*M. erythrini* (*s.s.*) and *M. isyebi*). The number of spines in the “pockets” of the genital atrium in *M. whittingtoni* n. sp. (34–47) overlaps with the ranges for *M. erythrini* (*s.s.*), *M. isyebi*, *M. omanae* and *Microcotyle* sp. ex *H. dactylopterus* [24].

The combination of characters for *M. whittingtoni* n. sp. previously mentioned in the remarks is also present in *M. isyebi*, *M. lichiae* and *Microcotyle* sp. ex *H. dactylopterus* [24]. Ariola [35] differentiated *M. lichiae* from other *Microcotyle* spp. predominantly by its large body size, asymmetrical haptor and concentric arrangement of the spines in the genital atrium. Additionally, *M. whittingtoni* n. sp. differs from *M. lichiae* by the shape of the haptor and the genital atrium; in fact Ariola [35] described a genital atrium with five rings of concentric spines in *M. lichiae*, unique among *Microcotyle* spp. Moreover, *M. lichiae* parasitizes a non-sparid host (Carangidae). More specimens of *M. lichiae* must be examined in order to determine the taxonomic status of this species. Regarding the outstandingly greater body length of *M. lichiae* (8000 *vs* 2719–4569 µm), this must be considered with caution as the size of polyopisthocotylean monogeneans is strongly related to host size, and thus not a reliable character in the taxonomy of polyopisthocotyleans [32]. Regarding *M. isyebi* and *Microcotyle* sp. ex *H. dactylopterus* [24], it is difficult to depict differential features for *M. whittingtoni* n. sp. other than parasitism in different hosts. The only clear differential morphological trait of *M. whittingtoni* n. sp. is the possession of more robust clamps, distinctly different from those in *M. isyebi* (ratio “c” sclerite maximum width/total clamp length, 0.100–0.146 *vs* 0.027–0.88). Another difference can be found in the posterior extremities of vitelline fields, always asymmetrical in *M. whittingtoni* n. sp., while *M. isyebi* includes some specimens with symmetrical fileds (21% according to the present study, see species description above). The data on *Microcotyle* sp. ex *H. dactylopterus* is limited, and some characters, such as clamp or egg morphology are not reported, therefore, until more specimens are analysed, the only evidence to differentiate these species is parasitism in different host species and molecular delineation ([24]; present study).

The specimens recorded by González González [36] in *D. dentex* from the Balearic Islands (identified as *M. erythrini*) belong to *M. whittingtoni* n. sp. due to the congruent morphology, host, and geographical distribution. The description by González González [36] agrees well with the description of the new species, except for the greater number of clamps (110–120 *vs* 60–78). However, the number of clamps in the specimen of the drawing and photograph in González González [36] has 60 clamps (figures 6 and 8 in [36]) in agreement with the description of the new species.

### Multivariate morphometric analysis

**Fig. 7:**
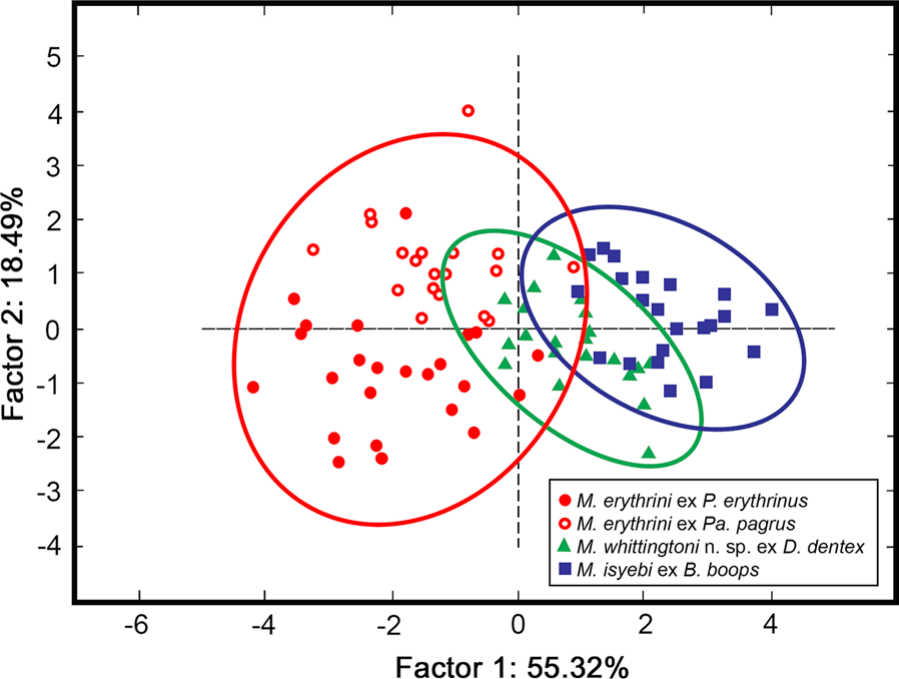
Plot of 87 specimens of *Microcotyle* spp. in the first plane of the PCA. Ellipses indicate 95% confidence intervals

The PCA using seven morphometric variables associated with body shape produced a plot of the 86 specimens (one extreme outlier was removed prior to analyses) in the first plane of the PCA showing the morphological variability between the species of *Microcotyle* from the Spanish Western Mediterranean (Fig. 7). The first two axes of the PCA explained 73.81% of the variation in the dataset. The first axis explained 55.32% of the variation and showed a separation between *M. erythrini* (*s.s.*) and *M. isyebi*, while *M. whittingtoni* n. sp. overlapped with the other two species. The specimens of *M. erythrini* ex *P. erythrinus* and *Pa. pagrus* showed a wider variation, whereas, for *M. whittingtoni* n. sp. ex *D. dentex* and *M. isyebi* ex *B. boops* the variation was lower. The first axis was positively correlated with the maximum body width (0.844), body length without the haptor (0.736), body width at level of the genital atrium (0.726) and body width at testis area (0.777), and negatively correlated with the length of the anterior haptor lobe (−0.856) and haptor length (−0.803). The second axis which was negatively correlated with body width at the level of the buccal suckers (−0.838) showed intraspecific separation between the specimens of *M. erythrini* ex *P. erythrinus* and ex *Pa. pagrus*.

## Discussion

No type-species was selected for the genus *Microcotyle* in the original definition by van Beneden & Hesse [4], which included the descriptions of two species, *M. donavini* and *M. erythrini* (also *M*. *canthari* and *M. labracis*, but these species currently belong to the genera *Neobivagina* and *Serranicotyle*, respectively). Later, Sproston [37] selected *M. donavini* as the type-species for the genus but at the time of the erection of the genus, these two species were the first morphological references. First descriptions of new *Microcotyle* species were based on vague morphological differences (mostly the number of clamps and testes [5, 38, 39]). Many more species have been described since then worldwide and several genera of the subfamily Microcotylinae have been erected, and *M. erythrini* has continued being considered valid [2, 3]. Recently, Bouguerche et al. [2] and Bouguerche et al. [3] provided molecular evidence that despite the validity of this species, several *Microcotyle* spp. from different host species have been wrongly identified as *M. erythrini* because of their morphological homogeneity. These authors referred to a *M. erythrini* complex of cryptic species and suggested that a molecular re-evaluation may reveal additional parasite diversity [3]. Caution must be taken in order to select representative specimens in perfect conditions of maturity, completeness and constitution. Whittington [40] stated that to separate monogeneans of a species complex with high levels of diversity “it is vital to ensure that there is a useful trail of high quality parasite material” for taxonomic studies, also stressing the importance of supporting the results with molecular genetic analyses. The present study shows that morphological differences between *M. erythrini* (*s.s.*) and similar species can be found: a new species of *Microcotyle* is described in *D. dentex*, together with the redescription of *M. erythrini* (*s.s.*) (including a new host record, *Pa. pagrus*) and a new geographical record of *M. isyebi* with additional morphological information, all supported by molecular evidence.

Molecular analyses of the *cox*1 gene showed clear differences between *Microcotyle* spp. distinctly separating the three species described here. Previous studies have suggested levels of intraspecific variation lower than 5% for species of mazocraeids and microcotylids (up to 5.6% and up to 4.5%, respectively, Yan et al. [41]; Mladineo et al. [42]). Based on *cox*1 sequences, *M. whittingtoni* n. sp. appears markedly distinct, since the genetic distance from the remaining congeners was higher than 10.8%; specifically, the two isolates ex *D. dentex* differed from *M. erythrini* (*s.s.*) by 10.8–13.5% and from *M. isyebi* by 14.4–15.2%. The available *28S* rDNA sequences for *Microcotyle* spp. are scarce as this region is not commonly used as a marker for interspecific differences.

We have delimited the valid morphological ranges of *M. erythrini* (*s.s.*) and similar species in sparids; in addition, we suggest the use of new diagnostic characters and morphological tools for assessment of multivariate patterns (e.g. PCA). One of the issues in defining the differential traits of similar species of microcotylids is to avoid abnormally wide morphological ranges by selecting only representative specimens: (i) not including specimens potentially belonging to other species (e.g. morphologically similar parasites from other host species not confirmed by molecular analysis); (ii) selecting morphologically optimal specimens (mature, unbroken, uncontracted, unstretched, not wrinkled and unfolded); and (iii) characterizing these specimens accurately to ensure that the diagnostic species-specific characters are properly described. More than 150 years after the original description of *M. erythrini*, numerous descriptions of this species from *P. erythrinus* and other hosts have provided extremely wide ranges of morphological information for this species, making it almost impossible to find differentiating features. By defining *M. erythrini* (*sensu stricto*) here, we aimed to characterize the species by much narrower morphological ranges only considering valid genetically tested specimens from confirmed hosts. Regarding the optimal specimen selection, only completely mature adults should be representative for standardized taxonomic descriptions. Worms with fully developed both male and female reproductive systems must be selected, as testes in young adults are early functional and vas deferens is full of sperm while no developed oocytes exist in the germarium. Also, the preservation and completeness of the specimens must be ensured.

Knowledge of the three-dimensional structure of the monogeneans in fresh preparations is crucial to understand the morphology of the specimens mounted in Canada balsam as under the coverslide they are represented in a two-dimensional view; knowledge of the natural shapes allows detecting possible folds and missing parts. For example, the haptor of *Microcotyle* spp. has a dorsal lobe and a ventral lobe (sometimes notched anteriorly), both with clamps; when the specimens are mounted (usually in ventral view), dorsal and ventral projections are folded and often the ventral lobe overlaps the haptor peduncle and/or the posterior end of the body (Fig. 1c). When measuring the haptor, this morphology must be considered in order to measure the body length and the haptor dimensions (see Fig. 1c, d). Moreover, when clamps are counted, possible gaps in the sequence of clamp frills (Fig. 1e, arrowhead) or possible missing pieces of haptor (Fig. 1e, arrow) must be considered, taking into account that the most distal clamps at the ends of the haptoral lobes are smaller.

In general, we recommend the revision and adequate counting of some discrete characters as the number of clamps or testes in the descriptions of several previously described species of *Microcotyle*, as some ranges are often abnormally high (e.g. 59–126 clamps for *M. visa* or 9–24 testes for *M. erythrini*, see [2, 3]). We must be particularly rigorous with this consideration as these traits are key in the species diagnoses of polyopisthocotyleans. For example, the number of clamps herein reported for *M. whittingtoni* n. sp. is 60–78, as the much higher range reported for *M. erythrini* of González González [36] in *Dentex dentex* (110–120) was not confirmed and the drawing and the photomicrograph show that their specimens had 60 clamps [36]. Regarding testes, it should be considered that they are flattened and stacked in at least two dorsoventral levels, so they must be detected and counted at different depth levels.

Some traditionally used morphological traits are intrinsically highly variable, and must be considered with extreme caution when used for taxonomy. Total length has been considered to characterize species such as *M. archosargi* and *M. lichiae* which are, in general, much larger than *M. erythrini* (*s.s.*) and similar species; however this trait is uncertain as monogenean sizes are known to be highly dependent on host size [32, 43]; e.g. Mendoza-Franco et al. [25] described smaller specimens of *M. archosargi*, lowering the range of body length to numbers that overlap with most of the similar species in sparid fishes (see Additional file 3: Table S3). In the case of *M. lichiae*, the original description was based on a single specimen and there are no data for its intraspecific variability. The number of spines in the genital atrium has been used more recently to differentiate species of *Microcotyle*; this character is often highly variable (e.g. for *M. isyebi* Bouguerche et al. [3] reported 154–267 *vs* 272–395 in the present study) and can depend on the condition of the specimen (e.g. incorrect fixation or genital atrium more or less evaginated) or discordances related to the observers. The “pockets” of the genital atrium (*sensu* Mamaev [44]; posterior small chambers), typical of the genus, have also provided taxonomic information. Mamaev [44] already indicated that the presence or absence of spines in these “pockets” was a good diagnostic character. For example, Bouguerche et al. [3] also stated that *M. isyebi* shared the presence of genital atrium “pockets” with the other *Microcotyle* species parasitic in sparid fishes (i.e. *M. archosargi*, *M. erythrini*, *M. isyebi* and *M. visa*) and not in species parasitic in fishes of other families (i.e. *M. donavini* and *M. lichiae*; these authors also listed *M. pomatomi* and *Microcotyle* sp. ex *H. dactylopterus* [24] but “pockets” are present in these species, see Remarks to *M. isyebi* above). “Pockets” are often not described and sometimes not clearly drawn (e.g. *M. pomatomi* [32]), as sometimes they can be unarmed or armed with a few spines [44]. Moreover, when the genital atrium is evaginated, chambers often become indistinguishable in ventral view. Their absence implies a different general structure of the genital atrium, a feature used for differentiation at the generic level within the subfamily Microcotylinae [30, 45]. Our last considerations of the traditionally used diagnostic traits refer to the dimensions of the soft muscular organs such as the pharynx or the genital atrium, both contractile and highly variable depending on the specimen, often mentioned in species descriptions (e.g. [2, 43]). All these soft organs can entail diagnostic evidence, but reliable differences should be outstanding, mostly referred to their volume or area, and if possible, relative to the specimen size.

The use of the correct tools and procedures can allow that the currently genetically differentiated species (*M. erythrini, M. isyebi* and *M. whittingtoni* n. sp.) become pseudocryptic with defining diagnostic characters or combinations of characters. When the morphometric data of individual worms was integrated in the PCA, the resulting components could not be useful to separate species but provided useful information on specimen groupings based on their shape. The results of the PCA in the present study illustrated that additional diagnostic information can be extracted from the general form of the worms, particularly regarding the relative dimensions and arrangement of the haptor and the remaining of the body. In view of this evidence, we suggest new diagnostic characters revealing previously unnoticed morphological differences: (i) haptor dimensions including anterior and posterior lobes (the larger values for haptor length to body length ratio and for anterior/posterior haptoral lobe length ratio differentiate *M. erythrini* (*s.s.*) from *M. isyebi* and *M. whittingtoni* n. sp.); (ii) thickness of the clamps (the higher ratio between “c” sclerite maximum width/total clamp length differentiates *M. whittingtoni* n. sp. from *M. isyebi* and *M. erythrini* (*s.s.*)); (iii) relative size and shape of spines of the “pockets” of the genital atrium (spines of the “pockets” in *M. whittingtoni* n. sp. appear to be longer and more curved than those of *M. isyebi* and *M. erythrini* (*s.s.*)); (iv) extension and symmetry of the posterior extremities of vitelline fields (posterior extremities of vitelline fields always asymmetrical in *M. whittingtoni* n. sp. *vs* occasionally symmetrical in *M. isyebi* and *M. erythrini* (*s.s.*)); and (v) shape of the tip of the abopercular filament of the egg; the solid (capitated or pointed) tips of the abopercular filaments differentiate *M. erythrini* (*s.s.*) from *M. isyebi* and *M. whittingtoni* n. sp. We propose that the region that can provide more taxonomic information is the haptor, taking into account its three-dimensional structure as an oval to fusiform (when pointed at both ends) “foot” holding a body perpendicularly inserted, directly or through a peduncle (Fig. 1c–e). In this way the total and relative haptor dimensions must include both lobes (anterior and posterior), and one of them is often unnoticed in mounted specimens because they fold over the body (see Fig. 1c, d). In fact, some authors have described the haptor of some *Microcotyle* species as triangular (e.g. [3, 26, 33, 34]) only referring to the lobe not folded over the body. In this way, *M. erythrini* (*s.s.*) can be defined by its relatively longer ventral lobe, the one that is usually unnoticed as it is adhered to the body in permanent mounts. As a note of caution, we must stress the need of examination of adult specimens only, as the relative dimensions of the haptor are known to change significantly during the development (see, for example Machkewskyi et al. [43]). The shape and size of the clamps also provides useful taxonomic information. These structures are usually described only as *Microcotyle*-type, and the width and length are provided (sometimes wrongly addressed, see Additional file 3: Table S3, Additional file 4: Table S4 and Fig. 1a for correct measuring). However, within this morphological description, some variations can be found. A more detailed study of clamp features can provide further taxonomic information. For example, the accessory sclerite (‘e’) is herein described as trifid or trident-shaped for all three species analysed, but it is mostly not described and not drawn, and the few authors drawing the sclerite represent it as single or lancet-shaped (e.g. [24, 46]). We also suggest that more attention should be paid to the thickness of the clamps: among the three species herein analysed, *M. whittingtoni* n. sp. shows noticeably thicker clamps; we explored this attribute through the width of the antero-lateral sclerite (‘c’) in absolute value and in relation to clamp length, as this region of the clamp appeared to be constant in all the specimens observed. The number of the spines in the “pockets” of the genital atrium is sometimes reported separately, but no specific information on the shape of these spines is usually found, except sometimes detailing that they are equal to those in the main chamber of the genital atrium [2, 3, 24]. Interestingly, these spines were observed to be longer than the spines of the main chamber in all three species herein described, and those in *M. whittingtoni* n. sp. were distinctly curved; the lack of information from other species prevented us to reach to further taxonomic conclusions, but we encourage the authors to provide specific information on the spines in the “pockets” in their descriptions of species of *Microcotyle*.

In the specimens of *Microcotyle* from the Spanish Western Mediterranean we observed some differences in the extension of the posterior extremities of vitelline fields (also including the extension of the caeca, as they accompany the vitellarium): extending into the haptor or peduncle in *M. erythrini* (*s.s.*) and into the haptor in *M. whittingtoni* n. sp. and prehaptoral in *M. isyebi*. However, this trait was not here suggested to characterize *M. isyebi* as according to the original description the posterior extension of the left caecum (and consequently the accompanying vitelline fields) extends into haptor “for a short distance” of the specimens from off Algeria [3]. This character may be dependent on the degree of contraction of the specimen, and therefore all specimens should be fixed and mounted in a similar way to be comparable. Other aspect related to the posterior extension of the vitelline fields of the vitellarium is their symmetry. We observed that the posterior extensions of the vitelline fields were always asymmetrical in *M. whittingtoni* n. sp., while in the other two species we found both specimens with symmetric and asymmetric vitelline fields. Gill polyopisthocotyleans show more or less distinct asymmetry related with the side of the gill filament they attach to [47, 48]; interestingly Bouguerche et al. [3] reported that left caecum (and consequently the accompanying vitelline fields) was longer in *M. isyebi*, while in all the species herein observed included specimens with both dextral or sinistral asymmetry.

Mamaev [44] described the eggs of *Microcotyle* spp. as two-filamented, with usually long opercular and shorter abopercular filament, but no further morphological details are normally provided in the species descriptions. The examination of the new specimens from the Spanish Western Mediterranean also revealed differential details regarding the eggs such as the different shapes of the end of the abopercular filament: solid (pointed or capitate) in *M. erythrini* (*s.s.*) (Fig. 4f) and hollow (bifid or cup-shaped) in *M. isyebi* and *M. whittingtoni* n. sp. (Figs. 5f, 6f). Other possible differential details were observed such as the type of connection between the egg and the opercular filament: abruptly connected in *M. erythini* (*s.s.*) and *M. isyebi* (Figs. 4f, 5f) and inserted through a gradual transition in *M. whittingtoni* n. sp. (Fig. 6f). This trait is not used for diagnosis in the present study as it requires a more standardized description. More detailed descriptions are recommended as this trait can be taxonomically useful and other authors, e.g. Sproston [37], have already reported interspecific differences regarding the egg shape. The information on this trait can be limited as the egg shape varies depending on the condition and presence of uterine eggs.

## Conclusions

The present study suggests new diagnostic morphological traits to differentiate *Microcotyle* spp. in Mediterranean sparids and shed light on the case of *M. erythrini* species complex changing its previously considered cryptic status. More detailed descriptions are recommended, including molecular data, preferably of more informative gene markers regarding the interspecific differences in the polyopisthocotyleans such as *cox*1 [41, 42, 49], but also *28S* rDNA sequences as they can provide useful complementary information. This study also shows that *M. erythrini* (*s.s.*) is not species-specific (even not genus-specific) to its hosts, as it parasitizes *Pa. pagrus* in addition to the type-host, *P. erythrini*; therefore, although the host species must continue as referential in the taxonomy of *Microcotyle* spp., a new host record does not necessarily mean a new species. However, further studies are needed in order to establish the morphological traits defining the microcotylids, especially for genera such as *Microcotyle*, with numerous species reported worldwide.

## Supplementary information

**Additional file 1: Table S1.** Mean genetic divergence (uncorrected p-distance in % and number of pairwise nucleotide differences in parentheses) estimated for the partial *cox*1 sequence pairs within- (along the diagonal, emboldened) and among species of *Microcotyle* (below the diagonal).

**Additional file 2: Table S2.** Pairwise nucleotide differences among species of *Microcotyle* for the partial *28S* rDNA sequences, including *Bivagina pagrosomi*.

**Additional file 3: Table S3.** Metrical data for *Microcotyle erythrini* (*sensu stricto*) and other species of *Microcotyle* in sparid fishes in the Mediterranean Sea and North-East Atlantic. Measurements are in micrometres expressed as ranges, except where a single value was provided.

**Additional file 4: Table S4.** Metrical data from descriptions of *Microcotyle* spp. similar to *M. erythrini* (*sensu stricto*) from Mediterranean non-sparid or non-Mediterranean fishes. Measurements are in micrometres expressed as ranges, except where a single value was provided.

## Data Availability

All data generated or analyzed during this study are included in this published article. The newly generated sequences were submitted to the GenBank database under the accession numbers MN816010-MN816021 (*cox*1) and MN814847-MN814850 (*28S*). The holotype and paratypes of *M. whittingtoni* n. sp., and vouchers of *M. erythrini* (*s.s.*) and *M. isyebi* were deposited in the Natural History Museum, London, UK (NHMUK.2019.12.10.1-NHMUK.2019.12.10.14); the remaining voucher material is deposited in the Parasitological Collection of the Cavanilles Institute of Biodiversity and Evolutionary Biology, University of Valencia, Spain (Acc. Nos. ICBiBE/PeMe2019, ICBiBE/PpMe2019, ICBiBE/BbMi2019 and ICBiBE/DdMw2019).

## Abbreviations

AHL: anterior haptor lobe length
BL: body length
BL-H: body length without haptor
CL: clamp length
CO: copulatory organ
CPS: Central South-East Pacific
CSW: ‘c’ sclerite width
CW: clamp width
EAS: Eastern Arabian Sea
HL: haptor length
MC: main chamber of the genital atrium
NS: North Sea
NWA: North-West Atlantic
NWP: North-West Pacific
P: small posterior chambers (“pockets” *sensu* Mamaev [44])
PHL: posterior haptor lobe length
SA: Southern Australia
SWP: South-West Pacific
WCA: Western-Central Atlantic
WM: Western Mediterranean
