# Supplementary material for: Considerations on the taxonomy and morphology of Microcotyle spp.: redescription of M. erythrini van Beneden & Hesse, 1863 (sensu stricto) (Monogenea: Microcotylidae) and the description of a new species from Dentex dentex (L.) (Teleostei: Sparidae)
Source: Parasit Vectors. 2020 Jan 31;13:45. doi: 10.1186/s13071-020-3878-9 (PMC7001340; doi:10.1186/s13071-020-3878-9)
Supplement: Supplementary file 1 — Additional file 1: Table S1. Mean genetic divergence (uncorrected p-distance in % and number of pairwise nucleotide differences in parentheses) estimated for the partial cox1 sequence pairs within- (along the diagonal, emboldened) and among species of Microcotyle (below the diagonal). [file 13071_2020_3878_MOESM1_ESM.docx]

**Additional file 1: Table S1.** Mean genetic divergence (uncorrected p-distance in % and the number of pairwise nucleotide differences in parentheses) estimated for the partial *cox*1 sequence pairs within- (along the diagonal, emboldened) and among species of *Microcotyle* (below the diagonal)

|  |  | **1** | **2** | **3** | **4** | **5** | **6** | **7** | **8** | **9** | **10** | **11** |
| --- | --- | --- | --- | --- | --- | --- | --- | --- | --- | --- | --- | --- |
| 1 | *M*. *whittintoni* ex *Dentex dentex* | **1.6 (6)** |  |  |  |  |  |  |  |  |  |  |
| 2 | *M*. *erythrini* ex *Pagrus pagrus* | 11.3 (43) | **0.8 (3.2)** |  |  |  |  |  |  |  |  |  |
| 3 | *M*. *erythrini* ex *Pagellus erythrinus* | 12 (40.3) | 1.6 (5.1) | **2.0 (6)** |  |  |  |  |  |  |  |  |
| 4 | *M*. *isyebi* ex *Boops boops* | 14.7 (53.1) | 13.3 (48.2) | 13.6 (45.7) | **0.9 (1.7)** |  |  |  |  |  |  |  |
| 5 | *M*. *visa* ex *Pagrus caeruleostictus* | 12.7 (48.3) | 11.3 (41,3) | 12.7 (43.6) | 8.5 (30.8) | **1.1 (1.7)** |  |  |  |  |  |  |
| 6 | *M*. *sebastis* ex *Sebastes schegeli* | 15.8 (60) | 13.3 (50.5) | 14.6 (50) | 9.2 (33.2) | 10.7 (30.8) | **̶** |  |  |  |  |  |
| 7 | *Microcotyle caudata* ex “*Sebastes inermis* species complex” | 14.9 (56.8) | 15.1 (57.3) | 15.9 (54.7) | 10.0 (36) | 9.2 (34.9) | 9.2 (34.6) | **1.5 (5.8)** |  |  |  |  |
| 8 | *Microcotyle* sp. ex *Sebasticus marmoratus* | 14.9 (56.5) | 13.8 (52.3) | 14.3 (49.3) | 9.9 (35.8) | 10.4 (39.3) | 8.2 (31) | 8.1 (30.8) | **0.0 (0)** |  |  |  |
| 9 | “*Paramicrocotyle*” sp.^a^ ex P*inguipes chilensis* | 13.9 (53) | 10.5 (40) | 11.2 (38.7) | 11.3 (41) | 11.2 (42.7) | 9.8 (37) | 11.7 (44.4) | 10.8 (41) | ̶ |  |  |
| 10 | *Microcotyle* sp. ex *Helicolenus dactiloptherus* | 15.3 (58) | 14.8 (56) | 16.2 (55.7) | 11.3 (40.8) | 10.9 (41.2) | 8.2 (43) | 12.5 (47.4) | 11.2 (42.5) | 11.9 (45) | **0.0 (0)** |  |
| 11 | *M*. *algeriensis* ex *Scorpaena notata* | 15.4 (58.5) | 15.7 (61) | 16.8 (58) | 12.5 (45.4) | 13.0 (49.7) | 13.5 (51) | 13.6 (51.8) | 12.6 (48) | 13.9 (53) | 4.6 (17.5) | **0.0 (0)** |

^a^Genus synonymized with *Microcotyle* [1,2]

**References**

1. Mamaev Yu L. The taxonomical composition of the family Microcotylidae Taschenberg, 1879 (Monogenea). Folia Parasitol. 1986;33:199–206.

2. Bouguerche C, Gey D, Justine JL, Tazerouti F. Microcotyle visa n. sp. (Monogenea: Microcotylidae), a gill parasite of Pagrus caeruleostictus (Valenciennes) (Teleostei: Sparidae) off the Algerian coast, Western Mediterranean. Syst Parasitol. 2019;96:131–47.
