# Supplementary material for: Considerations on the taxonomy and morphology of Microcotyle spp.: redescription of M. erythrini van Beneden & Hesse, 1863 (sensu stricto) (Monogenea: Microcotylidae) and the description of a new species from Dentex dentex (L.) (Teleostei: Sparidae)
Source: Parasit Vectors. 2020 Jan 31;13:45. doi: 10.1186/s13071-020-3878-9 (PMC7001340; doi:10.1186/s13071-020-3878-9)
Supplement: Supplementary file 2 — Additional file 2: Table S2. Pairwise nucleotide differences among species of Microcotyle for the partial 28S rDNA sequences, including Bivagina pagrosomi. [file 13071_2020_3878_MOESM2_ESM.docx]

**Additional file 2: Table S2.** Pairwise nucleotide differences among species of *Microcotyle* for the partial 28S rDNA sequences, including *Bivagina pagrosomi*

|  | **Species** | **1** | **2** | **3** | **4** | **5** | **6** | **7** | **8** | **9** | **10** | **11** | **12** |  |
| --- | --- | --- | --- | --- | --- | --- | --- | --- | --- | --- | --- | --- | --- | --- |
| 1 | *Microcotyle whittintoni* ex *Dentex dentex*^a^ |  |  |  |  |  |  |  |  |  |  |  |  |  |
| 2 | *Microcotyle isyebi* ex *Boops boops*^a^ | 2 |  |  |  |  |  |  |  |  |  |  |  |  |
| 3 | *Microcotyle erythrini* ex *Pagellus erythrinus*^a^ | 4 | 2 |  |  |  |  |  |  |  |  |  |  |  |
| 4 | *Microcotyle erythrini* ex *Pagrus pagrus*^a^ | 3 | 1 | 1 |  |  |  |  |  |  |  |  |  |  |
| 5 | *Microcotyle erythrini* ex *Pagellus erythrinus* (AM157221) | 4 | 2 | 0 | 1 |  |  |  |  |  |  |  |  |  |
| 6 | “Microcotylidae sp. M11” ex *Argyrosomus japonicus* (EF653386) | 2 | 1 | 0 | 0 | 0 |  |  |  |  |  |  |  |  |
| 7 | *Microcotyle sebastis* ex *Sebastes* sp. (AF382051) | 3 | 1 | 2 | 2 | 9 | 1 |  |  |  |  |  |  |  |
| 8 | *Microcotyle archosargi* ex *Archosargus rhomboidalis* (MG586867) | 1 | 0 | 1 | 1 | 1 | 1 | 0 |  |  |  |  |  |  |
| 9 | “Microcotylidae sp. M10” ex *Sebastes* sp. (EF653385) | 3 | 1 | 2 | 2 | 2 | 1 | 0 | 0 |  |  |  |  |  |
| 10 | *Microcotyle* sp. 1 SC-2018 (MH700256)^b^ | 6 | 4 | 5 | 5 | 5 | 4 | 3 | 2 | 3 |  |  |  |  |
| 11 | *Microcotyle arripis* ex *Arripis georgianus* (GU263830) | 8 | 6 | 7 | 7 | 7 | 6 | 5 | 5 | 5 | 4 |  |  |  |
| 12 | *Microcotyle* sp. 2 SC-2018 (MH700266)^c^ | 8 | 6 | 7 | 7 | 7 | 6 | 5 | 5 | 5 | 4 | 0 |  |  |
| 13 | “*Microcotyle*” sp. AKV-2016 (KU926692) | 116 | 114 | 115 | 115 | 115 | 94 | 112 | 80 | 108 | 115 | 112 | 112 |  |
| 14 | *Bivagina pagrosomi* ex *Sparus aurata (*Z83002) | 45 | 45 | 46 | 46 | 46 | 31 | 50 | 30 | 41 | 46 | 54 | 45 | 121 |

^a^Present study

^b^Host not reported
