# Supplementary material for: Considerations on the taxonomy and morphology of Microcotyle spp.: redescription of M. erythrini van Beneden & Hesse, 1863 (sensu stricto) (Monogenea: Microcotylidae) and the description of a new species from Dentex dentex (L.) (Teleostei: Sparidae)
Source: Parasit Vectors. 2020 Jan 31;13:45. doi: 10.1186/s13071-020-3878-9 (PMC7001340; doi:10.1186/s13071-020-3878-9)
Supplement: Supplementary file 3 — Additional file 3: Table S3. Metrical data for Microcotyle erythrini (sensu stricto) and other species of Microcotyle in sparid fishes in the Mediterranean Sea and North-East Atlantic. Measurements are in micrometres expressed as ranges, except where a single value was provided. [file 13071_2020_3878_MOESM3_ESM.docx]

**Aditional file 3: Table S3.** Metrical data for *Microcotyle erythrini* (*sensu stricto*) and other species of *Microcotyle* in sparid fishes in the Mediterranean Sea and North-East Atlantic. Measurements are in micrometres and are expressed as the ranges, except where a single value was provided.

| Parasite species | *M. erythrini* van Beneden & Hesse, 1863 (*s.s.*) | |  | *M. erythrini sensu* Parona & Perugia (1890)^c^ |  | *M. isyebi* Bouguerche, Gey, Justine & Tazerouti, 2019 | |  | *Microcotyle whittingtoni* n. sp. |  | *M. visa* Bouguerche, Gey, Justine & Tazerouti, 2019 |
| --- | --- | --- | --- | --- | --- | --- | --- | --- | --- | --- | --- |
| Host species | *P. erythrinus* (L.) | |  | *B. boops* (L.) and *P. acarne (Risso)* |  | *B. boops* (L.) | |  | *D. dentex* (L.) |  | *Pa. caeruleostictus* (Valenciennes) |
| Locality | off Montenegro, Central Mediterranean Sea | off Seté, France, Western Mediterranean Sea |  | off Genoa, Italy, Western Mediterranean Sea |  | off Bouharoun, Algeria, Western Mediterranean Sea | off Granada, Spain, Western Mediterranean Sea |  | off Balearic Island, Spain, Western Mediterranean Sea |  | off Bouharoun, Algeria, Western Mediterranean Sea |
| Source | [[1](#_ENREF_1)] | [[2](#_ENREF_2)]^b^ |  | [[3](#_ENREF_3)] |  | [[2](#_ENREF_2)] | as *M. erythrini* [[4](#_ENREF_4)] |  | as *M. erythrini* [[5](#_ENREF_5)] |  | [[6](#_ENREF_6)] |
| Sample size | (*n* =5) | (*n* =13) |  | (*n* =30) |  | (*n* =31) | (*n* =5) |  | (*n* =20) |  | (*n* =31) |
| BL | 2,300–2,700 | 720–1,390 |  | 4,000–5,000 |  | 2,370–4,750 | 2,316–2,614 |  | 1,600–5,160 |  | 1,910–4,620 |
| BW | 200–400 | 110–260 |  | – |  | 310–830 | 300–500 |  | 160–680 |  | 270–1,000 |
| BL-H | – | 400–860 |  | – |  | 1,750–3,981 | – |  | – |  | 1,300–400 |
| HL | – | 280–580 |  | – |  | 500–1,250 | – |  | 800–1,600 |  | 250–1,250 |
| NC | 100–110 | 82–132 |  | 90 |  | 54–102 | 90–100 |  | 60 (110–120)^e^ |  | 59–126 |
| CL^a^ | 25 | 48–80 |  | – |  | 10–31 | 25 |  | 30–60 |  | 12–35 |
| CW^a^ | 60 | 25–37 |  | 70 |  | 25–62 | 60 |  | 60–90 |  | 25–60 |
| SL | 40 | 40–85 |  | 70 |  | 40–75 | 33–43 |  | – |  | 30–85 |
| SW | 45 | 35–65 |  | 45 |  | 30–65 | 50–60 |  | – |  | 20–60 |
| PL |  | 30–55 |  | – |  | 25–50 | – |  | – |  | 20–55 |
| PW |  | 25–50 |  | – |  | 30–48 | – |  | – |  | 20–45 |
| NT | 16–19 | 9–24 |  | 16 |  | 13–29 | 16–19 |  | 28 (13–21)^e^ |  | 14–29 |
| TL | – | – |  | 28^d^ |  | 51–45 | – |  | – |  | – |
| TW | – | – |  | – |  | – | – |  | – |  | – |
| GAD | 250 | 232–287 |  | – |  | 175–375 | 250 |  | 540 |  | 120–600 |
| GAL | – | 85–140 |  | 70 |  | 65–175 | – |  | – |  | 50–130 |
| GAW | – | 75–110 |  | – |  | 45–120 | – |  | – |  | 45–95 |
| NSMC | – | 201–255 |  | – |  | 136–230 | – |  | – |  | 142–224 |
| SLMC | – | – |  | – |  | – | – |  | – |  | – |
| NSP | – | 31–34 |  | – |  | 18–38 | – |  |  |  |  |
| LSP | – | – |  | – |  | – | – |  |  |  |  |
| EL | – | – |  | 230 |  | 125–260 | 207–220 |  |  |  |  |
| EW | – | – |  | 90 |  | 45–95 | 85–90 |  |  |  |  |
| AFL | – | – |  | – |  | 125–260 | – |  |  |  |  |

*Abbreviations*: AFL, abopercular filament length; BL, body length; BW, body width; BL-H, body length without haptor; CL, clamp length; CW, clamp width; EL, egg length (without filaments); EW, egg width (without filaments); GAD, genital atrium to anterior extremity distance; GAL, genital atrium length; GAW, genital atrium width; HL, haptor length; NC, no. of clamps; LSP, length of spines in the “pockets”; NSMC, no. of spines in the main chamber of the genital atrium; NSP, no of spines in the “pockets”; NT, no. of testes; PL, pharynx length; PW, pharynx width; SL, sucker length; SLMC, spines length in main chamber of genital atrium; SW, sucker width; TL, testes length; TW, testes width.

^a^Clamp length and width represent the minimum and maximum measurements, respectively, reported by each author (see Fig. 1a for explanation of the clamp measures)

^b^Re-examined specimens of the personal collection of Professor Louis Euzet (see Bouguerche et al. [[2](#_ENREF_2)])

^c^Measurements not valid to be included in the description of species as they represent pooled data from two host species

^d^Measurement provided as diameter

^e^Measurements estimated from the drawing

**References**

1. Radujković BM, Euzet L. Faune des parasites de poissons marins du Montenegro (Adriatique Sud): Monogenes. Acta Adriat. 1989;30:51–135.

2. Bouguerche C, Gey D, Justine JL, Tazerouti F. Towards the resolution of the *Microcotyle erythrini* species complex: description of *Microcotyle isyebi* n. sp. (Monogenea, Microcotylidae) from *Boops boops* (Teleostei, Sparidae) off the Algerian coast. Parasitol Res. 2019;118:1417–1428.

3. Parona C, and A. Perugia, Res ligusticae, XIV, Contribuzione per una monografía del genere. Microcotyle. Ann. Museo Civico Storia Nat. Giacomo Doria. Genoa, Ser. 2a 1890;10:173–220.

4. López-Román R, Guevara Pozo D. Especies de la familia Microcotylidae (Monogenea) halladas en teleosteos marinos de la costa de Granada. Rev Ibérica Parasitol. 1973; 33:197–233

5. González González P. Parasitofauna branquial de *Dentex dentex* (Lineo, 1758) (Pisces; Sparidae). PhD thesis, University of Valencia; 2005. http://roderic.uv.es/handle/10550/15041

6. Bouguerche C, Gey D, Justine JL, Tazerouti F. *Microcotyle visa* n. sp. (Monogenea: Microcotylidae), a gill parasite of *Pagrus caeruleostictus* (Valenciennes) (Teleostei: Sparidae) off the Algerian coast, Western Mediterranean. Syst Parasitol. 2019;96:131–147.
