# Supplementary material for: Considerations on the taxonomy and morphology of Microcotyle spp.: redescription of M. erythrini van Beneden & Hesse, 1863 (sensu stricto) (Monogenea: Microcotylidae) and the description of a new species from Dentex dentex (L.) (Teleostei: Sparidae)
Source: Parasit Vectors. 2020 Jan 31;13:45. doi: 10.1186/s13071-020-3878-9 (PMC7001340; doi:10.1186/s13071-020-3878-9)
Supplement: Supplementary file 4 — Additional file 4: Table S4. Metrical data from descriptions of Microcotyle spp. similar to M. erythrini (sensu stricto) from Mediterranean non-sparid or non-Mediterranean fishes. Measurements are in micrometres expressed as ranges, except where a single value was provided. [file 13071_2020_3878_MOESM4_ESM.docx]

**Additional file 4: Table S4.** Metrical data from descriptions of *Microcotyle* spp. similar to *M. erythrini* (*sensu stricto*) from Mediterranean non-sparid or non-Mediterranean fishes. Measurements are in micrometres and are expressed as the ranges, except where a single value was provided.

| Parasite species | *M. omanae* Machkewskyi, Dmitrieva, Al–Jufaili & Al–Mazrooei, 2013 |  | *M. archosargi,* MacCallum, 1913 |  | *M. algeriensis* Ayadi, Gey, Justine & Tazerouti, 2016 |  | *M. sebastis* *sensu* Radujkovic and Euzet (1989) | |  | *M. pomatomi* Goto, 1899^b^ |  | *M. donavini* van Beneden & Hesse 1863 | | |  | *M. lichiae* Ariola, 1899 |
| --- | --- | --- | --- | --- | --- | --- | --- | --- | --- | --- | --- | --- | --- | --- | --- | --- |
| Host species | *Cheimerius nufar* (Valenciennes) |  | *Archosargus probatocephalus* (Walbaum) and *A. rhomboidalis* (L.) |  | *Scorpaena notata,* Rafinesque |  | *Helicolenus dactylopterus* (Delaroche) | |  | *Pomatomus saltatrix* (L.) |  | *Labrus bergylta* Ascanius |  | *Symphodus mediterraneus* (L.) |  | *Lichia amia* (L.) |
| Locality | off Shuweymiyyah and Sharbithat, Western Arabian Sea |  | off North-West Atlantic |  | off Bouharoun, Algeria Western Mediterranean Sea |  | off Montenegro, Central Mediterranean Sea | off Bouharoun, Algeria Western Mediterranean Sea |  | off Turkey, Eastern Mediterranean Sea |  | off Roscoff, France, North-East Atlantic |  | off Montenegro, Central Mediterranean Sea |  | off Genoa, Italy Western Mediterranean Sea |
| Source | [[1](#_ENREF_1)] |  | [[2-4](#_ENREF_2)] |  | [[5](#_ENREF_5)] |  | [[6](#_ENREF_6)] | [[5](#_ENREF_5)] |  | Sezen & Price in [7] |  | [8] |  | [[6](#_ENREF_6)] |  | [9] |
| Sample size | (*n* = 20) |  | (n=27) |  | (*n* = 35) |  | (*n* = 10) | (*n* = 20) |  | (*n* = ?) |  | (*n* = 10) |  | (*n* = 1) |  | (*n* = 1) |
| BL | 3,500–11,000 |  | 3,950–8000 |  |  |  | 2,500–3,300 | – |  | 2,401–2,850 |  | 4,000–5,000 |  | 4,000–5,000 |  | 8,000 |
| BW | 475–1,875 |  | 329–800 |  | 300–860 |  | 500–600 | – |  | 511–637 |  | 400–500 |  | 400–500 |  | 500 |
| BL-H | – |  |  |  | 1,900–4,300 |  | – | 410–3,800 |  | – |  | 3,000 |  | – |  | 1170 |
| HL | 1,250–3,225 |  | 1,050–2,600 |  | 450–1,040 |  | – | 570–1,200 |  | 889–1.011 |  | 1,000 |  | – |  | – |
| NC | 94–120 |  | 82–135 |  | 20–39 |  | 38–56 | 49–58 |  | 106–130 |  | 86 |  | 86 |  | 52 |
| CL^a^ | 26–55 |  | 36–87 |  | 40–78 |  | – | 40–69 |  | 33 |  | 33–50 |  | 33–50 |  | 100–300^d^ |
| CW^a^ | 70–100 |  | 59–95 |  | 48–85 |  | – | 42–74 |  | 54 |  | 42–87 |  | 42–87 |  | – |
| SL | 60–120 |  | 50–100 |  | 40–85 |  | 25–40 | 47–73^d^ |  | 39–49^d^ |  | 40 |  | 40 |  | 220 |
| SW | 40–95 |  | 44–104 |  | 39–76 |  | 40–45 | – |  | – |  | 65 |  | 65 |  | 64 |
| PL | 28–75^b^ |  |  |  | 50–100^c^ |  | – | 40–77 |  | – |  | 40 |  | – |  | 70 |
| PW | – |  |  |  | – |  | – | 50–69 |  | – |  | 50 |  | – |  | 51 |
| NT | 34–55 |  | 16–36 |  | 9–20 |  | 15–17 | 10–17 |  | 21–28 |  | 18–22 |  | 18–22 |  | 20 |
| TL | 100–220^b^ |  |  |  | – |  | – | – |  | – |  | 58–83^d^ |  | – |  | 50–83^d^ |
| TW |  |  |  |  | – |  | – | – |  | – |  | – |  | – |  | – |
| GAD | 1,624–4,800 |  | 329–469 |  | 110–400 |  | – | 270–520 |  | – |  | – |  | – |  | – |
| GAL | 125–214 |  | 100–180 |  | 77–175 |  | – | 95–160 |  | – |  | 250 |  | 250 |  | – |
| GAW | 127–193 |  | 80–180 |  | 82–130 |  | – | 102–150 |  | – |  | 175 |  | 175 |  | – |
| NSMC | 402^c^ |  |  |  | 68–162 |  | – | 104–307 |  | – |  | 117^c^ |  | – |  | – |
| SLMC | – |  |  |  | – |  | – | – |  | – |  | – |  | – |  | – |
| NSP | 44–48^d^ |  |  |  | 8–18 |  | – | 12–38 |  | – |  | 16–18 |  | – |  | – |
| LSP | 6–10 |  |  |  |  |  | – | – |  | – |  | – |  | – |  | – |
| EL | 260–300 |  |  |  | 215–257 |  | – | – |  | – |  | 200–225 |  | – |  | – |
| EW | 75–105 |  |  |  | 50–85 |  | – | – |  | – |  | 75–80 |  | – |  | – |
| AFL | – |  |  |  | – |  | – | – |  | – |  | 100 |  | – |  | – |

*Abbreviations*: AFL, abopercular filament length; BL, body length; BW, body width; BL-H, body length without haptor; CL, clamp length; CW, clamp width; EL, egg length (without filaments); EW, egg width (without filaments); GAD, genital atrium to anterior end distance; GAL, genital atrium length; GAW, genital atrium width; HL, haptor length; NC, no. of clamps; LSP, length of spines in pockets; NSMC, no. of spines in main chamber of genital atrium NSP, no of spines in pockets; NT, no. of testes; PL, pharynx length; PW, pharynx width; SL, sucker length; SLMC, spines length in main chamber of genital atrium; SW, sucker width; TL, testes length; TW, testes width.

^a^Clamp length and width represent the minimum and maximum measurements, respectively, reported by each author. See Fig. 1a for explanation of the clamp measuring

^b^Ranges extracted from minimum and maximum morphological data completed by Williams (1991), excluding Mediterranean report. Williams 1991, synonymized *M. pomatomi* with *M. austraiensis*, *M. debueni* and *M. temnodontis*

^c^Measurements estimated from the drawings

^d^Measurement was provided as diameter

**References**

1. Machkewskyi VK, Dmitrieva EV, Al-Jufaili S, Al-Mazrooei NA. *Microcotyle omanae* n. sp. (Monogenea: Microcotylidae), a parasite of *Cheimerius nufar* (Valenciennes) (Sparidae) from the Arabian Sea. Syst Parasitol. 2013;86:153–63.

2. MacCallum G. Further notes on the genus *Microcotyle*. Zool Jahrb. 1913;35:389–402.

3. Kritsky DC, Bakenhaster MD. Monogenoidean parasites of the gill lamellae of the sheepshead *Archosargus probatocephalus* (Walbaum) (Perciformes: Sparidae) from the Indian River Lagoon, Florida, with descriptions of four new species of *Euryhaliotrema* Kritsky & Boeger, 2002 (Dactylogyridae). Syst Parasitol. 2011;78:57–68.

4. Mendoza-Franco EF, Tun MCR, Anchevida AJD, del Río Rodríguez RE. Morphological and molecular (28S rRNA) data of monogeneans (Platyhelminthes) infecting the gill lamellae of marine fishes in the Campeche Bank, southwest Gulf of Mexico. ZooKeys. 2018;783:125.

5. Ayadi ZEM, Gey D, Justine JL, Tazerouti F. A new species of *Microcotyle* (Monogenea: Microcotylidae) from *Scorpaena notata* (Teleostei: Scorpaenidae) in the Mediterranean Sea. Parasitol Int. 2017;66:37–42.

6. Radujković BM, Euzet L. Faune des parasites de poissons marins du Montenegro (Adriatique Sud): Monogenes. Acta Adriat. 1989;30:51–135.

7. Williams A. Monogeneans of the families Microcotylidae Taschenberg, 1879 and Heteraxinidae Price, 1962 from Western Australia, including the description of *Polylabris sandarsae* n. sp. (Microcotylidae). Syst Parasitol. 1991;18:17–43.

8. Euzet L, Marc A: *Microcotyle donavini* Van Beneden et Hesse 1863 espèce type du genre *Microcotyle* Van Beneden et Hesse 1863. Ann Parasitol Hum Comp. 1963;6:875-86

9. Ariola V. Di alcuni trematodi di pesci marini. Boll Mus Zool Anat Comp Genova. 1899;4:1–10.
